# Unexpected Dynamic Binding May Rescue the Binding Affinity of Rivaroxaban in a Mutant of Coagulation Factor X

**DOI:** 10.3389/fmolb.2022.877170

**Published:** 2022-05-05

**Authors:** Zhi-Li Zhang, Changming Chen, Si-Ying Qu, Qiulan Ding, Qin Xu

**Affiliations:** ^1^ State Key Laboratory of Microbial Metabolism & Joint International Research Laboratory of Metabolic and Developmental Sciences, School of Life Sciences and Biotechnology, Shanghai Jiao Tong University, Shanghai, China; ^2^ Department of Laboratory Medicine, Ruijin Hospital, Shanghai Jiao Tong University School of Medicine, Shanghai, China; ^3^ Collaborative Innovation Center of Hematology, Shanghai Jiao Tong University School of Medicine, Shanghai, China

**Keywords:** coagulation factors, rivaroxaban, molecular dynamics simulations, molecular flexibility, structure-based drug design

## Abstract

A novel coagulation factor X (FX) Tyr319Cys mutation (Y99C as chymotrypsin numbering) was identified in a patient with severe bleeding. Unlike the earlier reported Y99A mutant, this mutant can bind and cleave its specific chromogenetic substrate at a normal level, suggesting an intact binding pocket. Here, using molecular dynamics simulations and MM-PBSA calculations on a FX-rivaroxaban (RIV) complex, we confirmed a much stronger binding of RIV in Y99C than in Y99A on a molecular level, which is actually the average result of multiple binding poses in dynamics. Detailed structural analyses also indicated the moderate flexibility of the 99-loop and the importance of the flexible side chain of Trp215 in the different binding poses. This case again emphasizes that binding of ligands may not only be a dynamic process but also a dynamic state, which is often neglected in drug design and screening based on static X-ray structures. In addition, the computational results somewhat confirmed our hypothesis on the activated Tyr319Cys FX (Y99C FXa) with an impaired procoagulant function to bind inhibitors of FXa and to be developed into a potential reversal agent for novel oral anticoagulants (NOAC).

## Introduction

**FIGURE 1:**
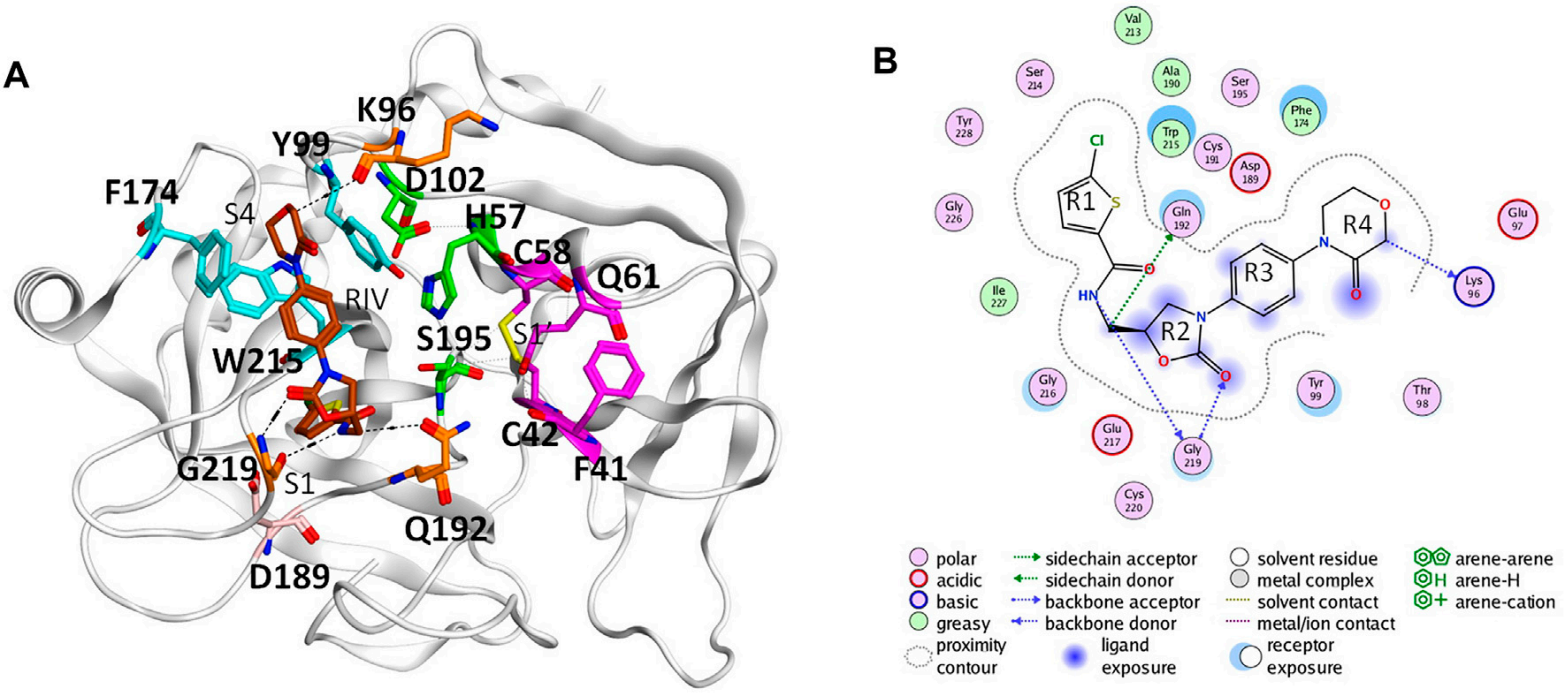
Binding of rivaroxaban (RIV) in the active site of coagulation factor X. **(A)** Three-dimensional structure from the Protein Data Bank (2W26). The carbon atoms of RIV are colored in brown, while those of the key residues in the S1 pocket and S1’ pocket are colored in magenta and those of the key residues in the S4 pocket are colored in cyan. Other residues concerning RIV-FX binding are colored in orange, with the catalytic triad in green and D189 in pink. **(B)** Two-dimensional plot of the binding of RIV with the interactions with the surrounding residues illustrated later. For convenience, the four rings of RIV are named as R1 to R4 from the chlorothiophene moiety to the morpholinone moiety.

In the coagulation cascade, the key position of coagulation factor X (FX) where the intrinsic and the extrinsic pathway merge into the common pathway makes it an ideal target to develop anticoagulants (Davie et al., 1991; Al-Obeidi and Ostrem, 1999; Lee and Player, 2011). Advances in crystallography have boosted the screening and design of synthetic inhibitors targeting on activated FX (FXa), which successfully resulted into novel oral anticoagulants (NOACs) approved by FDA, such as apixaban, rivaroxaban, and edoxaban (Perzborn et al., 2011; Wong et al., 2011; Wang et al., 2016). The X-ray structures of the FXa-inhibitor complexes showed that this type of anticoagulant can directly bind to the S1 pocket and S4 pocket at the same time (Maignan et al., 2003; Nazare et al., 2005). The former is a conserved pocket in the catalytic serine protease domains of coagulation factors, which accommodates the P1 residue of the peptide bond to be cleaved. The conserved Asp189 deep in the bottom of the pocket could provide strong electrostatic interaction with substrates and inhibitors (Katz et al., 2000; Hedstrom, 2002). At the same time, different from other coagulation factors with a serine protease domain, its distinctive hydrophobic S4 pocket provides an ideal target site for inhibitors to bind specifically (Wang et al., 2012). For example, the widely used NOAC rivaroxaban (RIV) has its chlorothiophene moiety (R1) and the morpholinone moiety (R4) binding to the S1 and S4 pocket of FXa, respectively (Roehrig et al., 2005) (Figure 1).

As the first approved NOAC, rivaroxaban has been increasingly used in clinical practice for treatment or prevention of thromboembolism. However, patients taking NOACs may present with major bleeding or need for management of an urgent unplanned bleeding challenge, and the best method to stop bleeding is the use of NOACs reversal agents to bind excessive NOACs (Kaatz et al., 2012; Samama, 2013; Samama et al., 2013). Until 2018, only one reversal agent for apixaban and rivaroxaban was approved by the Food and Drug Administration (FDA) (Escolar et al., 2017; Sartori and Cosmi, 2018; Carpenter et al., 2019), which is a recombinant FXa named andexanet alfa. However, in the ANNEXA-4 study, thrombotic events occurred in 18% of the patients in the safety population (Connolly et al., 2016), which is likely related to the binding of andexanet alfa to tissue factor pathway inhibitor (TFPI) (Ersayin et al., 2017). In the same way, different mutants of FXa were in development for more efficient reversal agents. For example, Verhoef et al. (2017) designed recombinants of FXa with either point mutation in the S4 pocket or fragment modifications on the 99-loop and compared their potentialities as reverse agents for NOACs with combined computational and biochemistry approach.

In addition to the fancy crystal structures of coagulation factors, molecular dynamics (MD) simulation also provided important understandings of the dynamics in FXa and its complex with different binding substrate/ligands. Daura et al. (2000) simulated the catalytic domain of FXa in aqueous solution and suggested possible hydrogen bonding of the active site residues with a substrate or inhibitor. Later, more simulations unveiled possible conformational changes in zymogen activation (Camire, 2002; Venkateswarlu et al., 2002) and in conformational transitions of open/closed states of the binding pocket (Singh and Briggs, 2010; Wang et al., 2012). MD simulations also suggested the importance of flexibilities to the catalytic activity impacted by mutations in the S4 pocket and S1 pocket (Abdel-Azeim et al., 2014) as well as the N-terminus of the serine protease domain (Li et al., 2019). The dynamics lying behind drugs targeting the S1 and S4 binding sites of FX were also implemented, such as edoxaban, betrixaban (Du et al., 2019), and rivaroxaban (Qu et al., 2019).

In this work, a novel FX Tyr319Cys mutation identified in a patient with severe bleeding diathesis was reported, whose activity for prothrombin activation is completely impaired but the cleavage on specific chromogenetic substrate is normal. A molecular model of the activated FX (FXa) with corresponding Y99C mutation in complex with rivaroxaban was then analyzed by molecular dynamics simulations. Unlike the Y99A mutant in which RIV is quickly released, this Y99C mutant is more like F174A and can have RIV dynamically bound with multiple patterns and unexpected strong affinity (Qu et al., 2019; Qu and Xu, 2019). Therefore, we presume that the activated Tyr319Cys FX (Y99C FXa) mutant may not have a procoagulant function but may have the ability to bind the NOAC rivaroxaban and the potential to be developed into a novel reversal agent.

## Materials and Methods

### Blood Sampling

The peripheral blood was collected *via* venipuncture into tubes containing sodium citrate (final concentration 0.38%), followed by double centrifugation at 3,000 g for 15 min to obtain platelet-poor plasma (PPP). Normal pooled plasma (NPP) was prepared from 30 healthy donors. The study was approved by the Institutional Review Board of Ruijin Hospital. All related individuals gave their informed consent to participate.

### Hemostatic Assays

The FX clotting activity (FX:C) was measured using the activated partial thromboplastin time (aPTT) and pro-thrombin time (PT) pathway-based coagulation function assays on the ACL-TOP automatic coagulometer (Instrumentation Laboratory). The plasma FX was activated to activate FX (FXa) by Russell’s viper venom (RVV-X) (Haematologic Technologies Inc., Essex Junction, VT, United States), and its enzymatic activity was determined using specific chromogenic substrate S2765 (Hyphen-Biomed, Neuville-Sur-Oise, France). The antigen level of FX (FX:Ag) was measured using an enzyme-linked immunosorbent assay (ELISA) kit (Enzyme Research Laboratories, South Bend, United States).

### Genetic Analysis of F10

Genomic DNA was extracted from peripheral whole blood using the QIAamp DNA blood purification kit (Qiagen, Hilden, Germany). The coding sequences and flank regions of F10 gene were amplified by polymerase chain reaction and sequenced. The primers used are listed in Table 1.

**TABLE 1:** Primers for *F10* amplification.

Exon	Forward 5’-3’	Backward 5’-3’
Exon 1	GTG​GTC​ACT​CCC​CTG​CCT​CG	TGC​TGT​GCC​CCT​CGT​CCT​G
Exon 2	TGA​GGG​TGA​CCA​GAG​CTT​TT	CTG​TGG​CCT​GAG​CTC​CTT​AC
Exon 3	TAA​GAT​GAC​TGA​AGC​CAC​AT	CTA​TTA​TGG​AAA​CAC​CCT​GA
Exon 4	GAA​ACA​GCT​TGC​AGA​CTC​CAG	CTT​CAG​GGG​CAT​CTG​ATC​T
Exon 5	CCT​TTG​CTC​AAC​CCA​ATG​GC	TGG​TGT​CAC​TGT​TAC​CTG​CC
Exon 6	TAT​GGG​GAG​CCT​CTC​TCT​GT	CAG​GTG​GTC​TCT​CCA​GCA​G
Exon 7	TGG​CAC​AGG​CAG​AGA​AAA​GA	CCT​CTG​TGA​AAT​GCC​CCT​AA
Exon 8	GAT​GTG​CGA​GAG​CAT​GTC​C	GGC​AAT​CGA​GAG​ACA​AAC​CA

### Modeling

The original molecular model of wild type FXa-RIV complex was based on the structure with ID as 2W26 in the Protein Data Bank (Roehrig et al., 2005), in which only the heavy chain of FXa, the ions, and crystallographic waters close to it were kept so as to reduce the size of the system (Abdel-Azeim et al., 2014). Tyr99 was selectively mutated into Cys and Ala by PyMOL as the initial mutant models. PyMOL (Schrodinger, 2015) was also used for visualization of the 3-D structures for both the initial models and the representative conformations from the simulations given later, while the 2-D plot of the RIV binding to critical residues was obtained by ProteinPlus (Fricker et al., 2004).

### Molecular Dynamics Simulations

The procedure of molecular dynamics simulations is fairly same as that of our earlier studies (Li et al., 2019; Qu et al., 2019; Qu and Xu, 2019). The package of GROMACS5.1.2 was used for the molecular dynamics simulations (Abraham et al., 2015). The force field for FXa was using CHARMM36 (Klauda et al., 2010), that for water was TIP3P (Jorgensen et al., 1983), and the parameters of RIV bound to the protein were generated by CHARMM General Force Field (CGenFF) (Vanommeslaeghe et al., 2010; Vanommeslaeghe et al., 2011; Vanommeslaeghe et al., 2012). The two calcium ions in 2W26 were retained in the topology and described by the default parameters of CHARMM36. A total of four pairs of disulfide bridges (Cys22-Cys27, Cys42-Cys58, Cys168-Cys182, and Cys191-Cys220) were defined as linked in the topology. The protonated states of all the residues were determined by H++ (Anandakrishnan et al., 2012) at PH value = 7.0 with the water model of TIP3P followed by manual checking (sequence of Y99C mutant is shown as an example in Supplementary Figure S1), from which His83 was set as the “HID” in the topology while other histidines (His57, His91, His145, and His199) were set as the “HIE.” The models were solvated in a cubic SPC216 water box (Berendsen et al., 1981) with the dimensions as 6.90 nm × 6.96 nm × 5.91 nm. The solvated systems were neutralized by adding five chloride ions, with the total number of atoms about 28,200. These systems were then energy-minimized by the steepest descent algorithm until the maximum force was lower than 1,000.0 kJ/mol/nm and then equilibrated with 100 ps NVT ensemble at 310 K and 100 ps NPT ensemble at 310 K and 1 atm, where the Nosé–Hoover weak coupling algorithm (Hoover, 1985) was used for temperature maintenance and the Parrinello–Rahman barostat methodology (Parrinello and Rahman, 1981) was used to keep the pressure. At the same time, the particle mesh Ewald (PME) (Perera et al., 1995) method was used for long-range electrostatic interactions and the Linear Constraint Solver algorithm (LINCS) (Hess et al., 1997) was used to allow the integration step as 2 fs. After the NVT and NPT equilibrations, at least 200 ns further simulations were performed and collected three times under the same NPT condition.

### Calculation of the Binding Free Energy

The g_mmpbsa (Kumari et al., 2014) package of GROMACS was used to calculate the binding free energy between RIV and FXa using the Molecular Mechanistic Poisson–Boltzmann Surface Area (MM-PBSA) method. In this work, the molecular mechanistic (MM) energies were only considered the electrostatic interactions and the van der Waals interactions. The solvation energies including the polar interactions were calculated by the Poisson–Boltzmann method and the nonpolar interactions were empirically estimated by the exposed area (SASA) using the surface tension coefficient γ = 0.0072 kcal/(mol•Å2). The entropy contribution was not included in this calculation for simplicity (Chen et al., 2013). In this way, the total binding free energy is:

$$\Delta {\text{G}}_{\text{bind}}=\Delta {\text{E}}_{\text{vdw}}+\Delta {\text{E}}_{\text{coulomb}}+\Delta {\text{G}}_{\text{polar}}+\Delta {\text{G}}_{\text{nonpolar}}$$

Due to the highly dynamic behavior of RIV observed in the mutants, we performed the calculation of the binding free energy in different methods for different purposes. For the overall comparison between the binding energies between the three systems (WT, Y99C, and Y99A), we concatenated the three trajectories of 200 ns MD simulations in each system to obtain a sampling set of 600 ns in total and picked out frames by intervals of 0.2 ns (3,000 frames in total for each system) for further calculation on the binding affinity of RIV. For calculation of the binding energies of the multiple binding modes in the Y99C mutant, the 3,000 frames were clustered as described later, and the frame sets of the top three clusters were collectively used for calculation of the RIV binding energy. Similarly, the MM-PBSA calculations were also applied to the frame sets of the top three clusters obtained separately from each 1,000 frames of the 200 ns trajectories of the Y99C mutant so as to explore the possible influence from different samplings between the three repeats.

### Analyses on Simulation Trajectories

The g_rms and g_rmsf package of GROMACS were used for calculation of the root-mean-square deviations (RMSDs) of selected atoms and the root-mean-square fluctuations (RMSFs) of the backbone atoms of the selected residues from Asn92 to Ile103 as the 99-loop, respectively. The RMSD of RIV or of the 99-loop backbone was also used for clustering the RIV binding modes or the conformations of the 99-loop, respectively, by the g_cluster package using the method illustrated by Daura et al. (1999). The frames for clustering were obtained from the trajectories with 0.2 ns interval. The cut-off for clustering was empirically selected as 0.17 nm based on the results of all the three systems. The package of “hydrogen bonds” in VMD (visual molecular dynamics) (Humphrey et al., 1996) was used to count possible hydrogen bonds between RIV and FXa, where any time a donor atom and an acceptor atom is less than 3.5 Å in distance and the angle of donor-H…acceptor is less than 30°, a hydrogen bond is counted. For example, when a residue forms two hydrogen bonds with RIV at the same time, no matter with side chain or backbone, the frequency is counted twice, and its overall occupancy is the sum of frequency involving this residue divided by the sum of frames from all three repeats of simulations. At last, the dynamic distribution of the R4 group of RIV was visualized by the positions of the C3 atom on it for simplicity.

## Results

### Clinical Results

**FIGURE 2:**
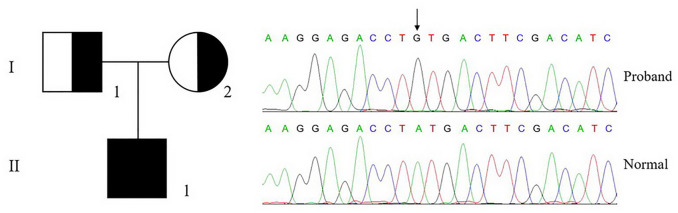
Genetic analysis of proband and pedigree. The genetic analysis shows that the proband (II-1) carries a homozygous c.956a > g, p.Tyr319Cys mutation in F10, which is inherited from his parents.

The genetic analysis of a patient (II-1) with severe bleeding diathesis identified a homozygous c.956a > g mutation in F10, which was inherited from his heterozygous father (I-1) and mother (I-2), who carries only one copy of this mutation on this allele (Figure 2).

This mutation is leading to a p.Tyr319Cys mutation in FX or the Y99C mutation in FXa by chymotrypsin numbering. The plasma FX antigen levels (FX:Ag) of both the proband (II-1) and his parents (I-1 and I-2) are almost normal as about 100% of normal control. The clotting time was prolonged in both APTT and PT tests. The FX clotting activity (FX:C) was determined by activated partial thromboplastin time (APTT) and prothrombin time (PT), which was completely impaired in the proband as 1.1%–1.4% of normal control, and more importantly, lowered to around 50% in the heterozygotes. However, the proteolytic activity to the chromogenic substrate S2765 was almost unchanged, all around 100% of the normal control (Table 2). It is reasonable to assume an intact active site in the Y99C mutant, at least for small substrates/inhibitors.

**TABLE 2:** Clotting function and genetic profile of the pedigree.

	FX:C (%)—Clotting activity		FX:C (%)—Chromogenic activity	FX:Ag (%)	*F10* mutation
	APTT	PT			
Proband	1.1	1.4	98.0	97.8	Tyr99Cys
I-1	55.2	49.8	101.2	103.9	Tyr99Cys (Het)
I-2	52.7	51.6	99.5	97.3	Tyr99Cys (Het)
Reference	50–150	50–150	50–150	50–150	

APTT, activated partial thromboplastin time; PT, pro-thrombin time.

### Comparison Between the Rivaroxaban Bindings in Different FXa Systems

Combining the three repeats of 200 ns simulations into one trajectory, the overall binding free energies of RIV to wild type (WT), Y99C, and Y99A mutants were calculated by MM-PBSA, with the contributions from van der Waals interactions, electrostatic interactions, and polar and nonpolar interactions with solutions compared in Table 3. From the total binding energies, it was found that the overall binding of RIV–Y99C (−83.018 ± 1.770 kJ/mol) is surprisingly even stronger than that of wild type FXa (−59.450 ± 2.011 kJ/mol), while the binding in Y99A mutant is much weaker. The unexpected stronger binding in Y99C is mainly from van der Waal interactions, where the binding is improved from −132.144 ± 2.761 to −162.162 ± 2.531 kJ/mol, although the polar interactions with solutions offset this improvement by about 10 kJ/mol. On the other side, the binding of RIV to Y99A is weakened in all the four terms of interactions since RIV was released from the binding site to the solution in a major part of the simulations.

**TABLE 3:** Comparison between the binding free energies of RIV in the wild type (WT), Y99C, and Y99A mutants of coagulation factor X.

Energy (KJ/mol)	WT	Y99C	Y99A
van der Waal energy	−132.144 ± 2.761	−162.162 ± 2.531	−75.749 ± 2.435
Electrostatic energy	−28.991 ± 1.134	−28.291 ± 0.924	−16.009 ± 0.778
Polar solvation energy	115.871 ± 2.565	124.552 ± 2.347	72.579 ± 2.596
Nonpolar solvation energy	−14.250 ± 0.294	−17.069 ± 0.256	−8.598 ± 0.266
Total binding energy	−59.450 ± 2.011	−83.018 ± 1.770	−27.605 ± 2.513

Residual contributions to the RIV binding were also analyzed. As shown in Supplementary Table S1, in wild type, only the three key residues of S4 pocket: Tyr99, Phe174, and Trp215 contribute more than 2 kJ/mol to the binding of RIV, and in all these three residues, Trp215 may contribute the most, same as our earlier analyses (Qu and Xu, 2019). In the Y99C mutant, the side chain of residue 99 is changed from aromatic tyrosine into a polar but much shorter cysteine. It was expected that the aromatic cage of the S4 pocket is damaged and its hydrophobic interactions with RIV would be weakened, as observed in Y99A. However, although the contribution from Cys99 (−2.916 ± 0.100 kJ/mol) is relatively a little lower than Tyr99 in wild type (−3.205 ± 0.149 kJ/mol), the stronger interaction from Trp215 (−8.497 ± 0.174 kJ/mol in Y99C versus −6.453 ± 0.178 kJ/mol in wild type) resulted in even stronger binding of RIV. In addition, another residue also contributes more than 2 kJ/mol to RIV binding: Val213 turns to contribute −2.204 ± 0.076 kJ/mol. This residue is close to Trp215 but on the edge between the S4 and S1 pocket, which may suggest more fluctuations in RIV binding pose in Y99C. On the other hand, in the Y99A mutant, the contributions from all the three key residues are much lowered, partly because RIV is released from the binding site. However, even in our earlier study where only frames before RIV were released and used from binding free energy calculations, the contributions of Ala99 and Trp215 are both about 5 kJ/mol lower than those in wild type. The importance of residues in the three trajectories are quite different, which is consistent with the observation that RIV is released from the binding pocket and may transiently bind to different positions on the surface of FXa.

**FIGURE 3:**
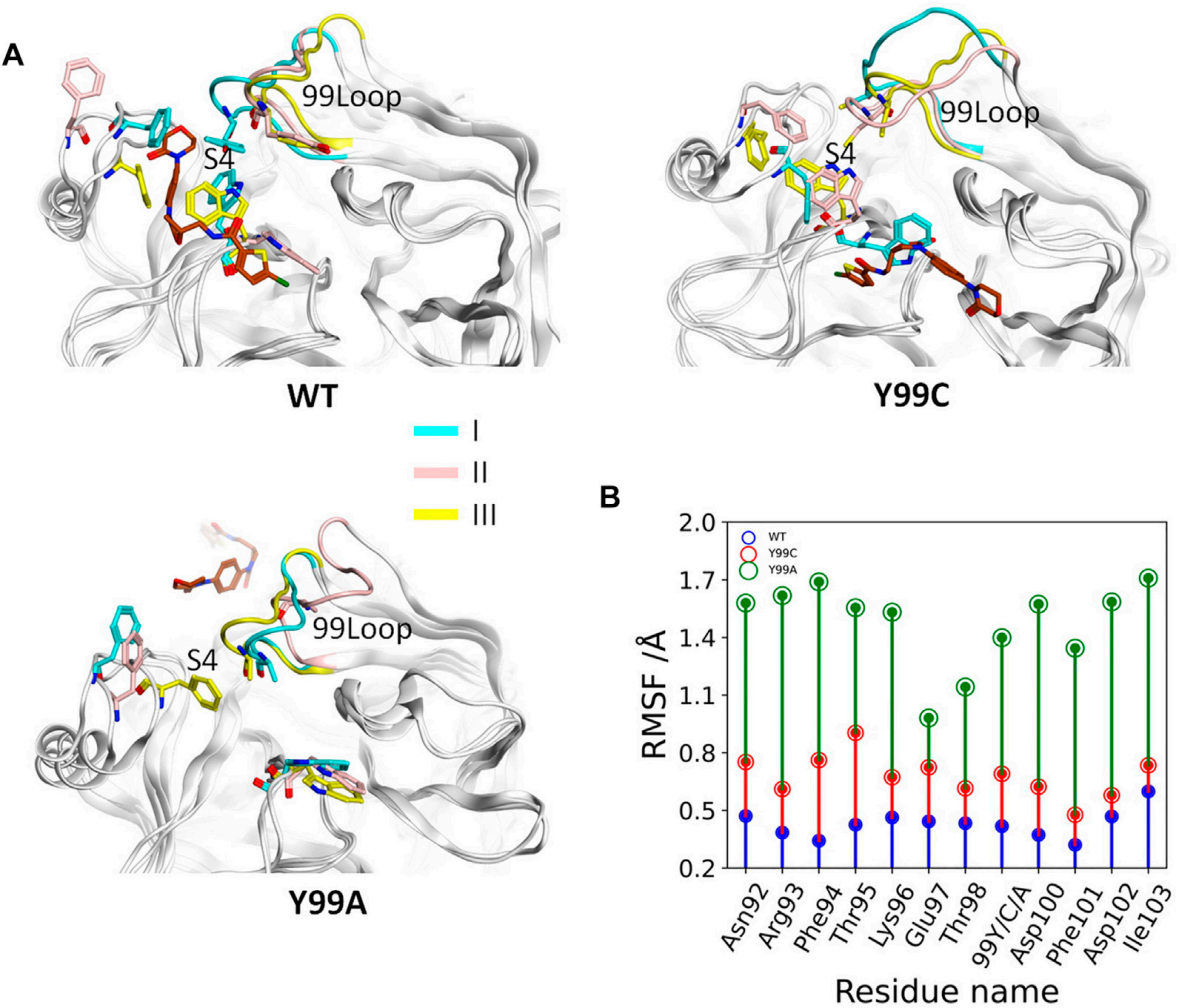
Comparison of the 99-loop fluctuation in WT, Y99C, and Y99A mutant of coagulation factor X. **(A)** Representative conformations of the top cluster from the three trajectories in three systems, with RIV (brown), the backbone of 99-loop, and the residues 99, Phe174, and Trp215 shown and colored. **(B)** Time evolution of average RMSF of 99-loop residues in WT (blue), Y99C (red), and Y99A (green) mutant of FX.

According to the binding free energy analyses, the main difference between the RIV binding in wild type and the mutants comes from the hydrophobic interactions, where deformation of the S4 pocket was expected to be caused by the mutations (Qu and Xu, 2019). Comparisons of the global RMSF clearly show that the difference in backbone flexibilities mainly happens in the 99-loop, where the flexibility in Y99A mutant is much higher than that in the other two systems (Supplementary Figure S2). As compared in Figure 3B, the average RMSF of all the residues on the 99-loop is a little increased in the Y99C mutant from about 0.5 Å to about 0.8 Å, but greatly increased to about 1.5 Å in the Y99A mutant. More details of each trajectory are shown in Supplementary Figure S3. The RMSD and RMSF of the 99-loop in wild type are not only stable, but also similar in all three trajectories. In Y99C, only those of trajectory III are as stable as wild type. In trajectory II, RMSD of the 99-loop is increased to 3 Å after 100 ns of simulations, resulting in a little higher RMSF as 0.5–0.8 Å. However, in trajectory I, the value of RMSD is quickly increased to about 3 Å after a short time, and part of the 99-loop (Phe94) could have RMSF as high as 1.8 Å. Consistently, the cross-correlation analysis of Cα atoms of the Y99C mutant indicates anti-correlated movements of the 99-loop with the two segments surrounding the S4 pocket, while no notable correlations are detected with other regions (Supplementary Figure S4), suggesting that the S4 pocket is not totally deformed. In all the three trajectories of Y99A, RMSD is quickly increased and fluctuates acutely from 2.5 to 4.5 Å, and RMSF varies from 1.0 to 2.0 Å, which is quite different both between the residues and between the trajectories. The representative structure of the top cluster of the trajectories is shown in Figure 3A. In two trajectories of WT and Y99C, the orientations of the side chains of Y99, Phe174, and Trp215 are roughly similar around the S4 pocket, and the backbone of the 99-loop is in comparable positions. However, in the trajectories II of WT and I of Y99C, the side chain of Trp215 flips to the side of S1, and the side chain of Phe174 in the former and the backbone of the 99-loop in the latter deviates away, leaving the S4 pocket open. At last, in Y99A, the side chains of Trp215 flip to the side of the S1 pocket in all three trajectories, while Phe174 and the 99-loop are rather deviated from the S4 pocket, which is too loose to bind RIV stably.

**FIGURE 4:**
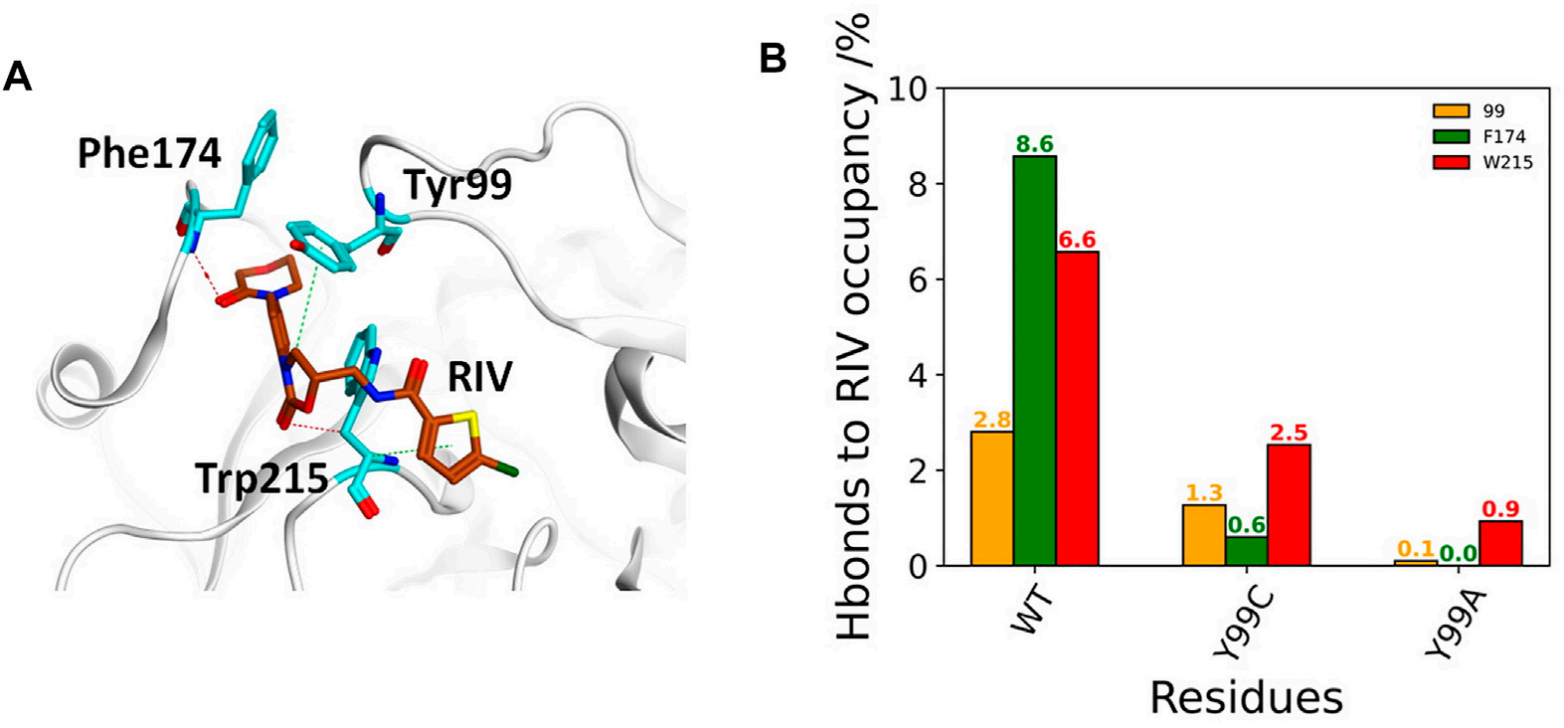
Comparison of FX residue hydrogen-bonding with RIV in WT, Y99C, and Y99A mutant. **(A)** Illustration of the H-bonding (red dash) and H-π interaction (green dash) of RIV with the key residues in the S4 pocket of the wild-type system, where RIV has the carbon atoms colored in brown while those of the residues were colored in cyan. **(B)** Different H-bonding occupancies of the three key residues Y/C/A99 (in orange), F174 (in green), and W215 (in red) with RIV.

The deformation in the S4 pocket may affect not only the hydrophobic interactions but also the electrostatic interactions between RIV and FXa, as illustrated in Figure 4A. The total occupancies of H-bonds involving the key S4 pocket residues in all simulations are summarized in Figure 4B. In the stable S4 pocket of wild type FXa, RIV may form H-bonds with Tyr99, Phe174, and Trp215 as high as 2.8%, 8.6%, and 6.6% of the time, respectively. It is surprising that rearrangement of the S4 pocket induced by the Y99C mutation only reduces the possibility for RIV to form H-bonds with the residue 99 and Trp215 by about 60%, although the most stable H-bonds with Phe174 in WT are almost lost. At last, the occupancies of H-bonds with Trp215 is greatly reduced and hardly found with Ala99 or Phe174. The residues possibly H-bonding with RIV with more than 2% of the simulation time are all listed in Supplementary Table S2. In all three systems, the residues Gln192, Gly216, and Gly219 at the entry of the S1 pocket are always of the top ones, and their occupancies are the only ones more than 10% in both WT and Y99C. In WT, the next important residues with the occupancies higher than 5% are Phe174 and Trp215 in the S4 pocket. However, their occupancies are much lowered in Y99C, with only Trp215 as low as 2.5%. Instead, residues around the catalytic pocket, such as Ser195, Lys96, and His57, have elevated occupancies as 8.2%, 5.8%, and 5.3%, respectively. This difference may suggest a shift of RIV binding poses. In addition to increased frequency of H-bonding between RIV and His57, the introduced Cys99 may also proximate to His57 and interfere with its H-bond with Asp102 considering the high flexibility of the 99-loop or even act as a nucleophile as in a cysteine protease. However, at least in this RIV-bound model, the time evolution of the distance between H57 [ND1] and C99 [HG1] shows that the average distances (9.7, 8.4, and 6.4 Å) in the three trajectories of Y99C are too far for the proton transfer between these two residues accompanied with a possible nucleophilic attack by Cys99 (Supplementary Figure S5). At last, almost all residues in Y99A have relatively lower possibility to form H-bonds with RIV, which is consistent with the fact that RIV is rather released. However, Lys62 on the edge of the S1’ pocket is the only residue with occupancy increased to 8.1%, which may suggest that the half-released RIV is easier to bind to the half-exposed S1’ pocket, similar to what we discussed on the F174A mutant earlier (Qu et al., 2019).

### Analyses on the Dynamic Binding in the Y99C Mutant

As compared previously, the binding of RIV in the Y99C mutant is neither as stable as in wild type FXa nor as totally released as in Y99A. The deformed S4 pocket, fluctuated 99-loop, and different interactions may suggest a dynamic binding in Y99C. Therefore, all the simulations of the Y99C mutant were combined together for clustering on the RMSD of RIV so as to figure out its most possible binding poses. MM-PBSA calculations were applied to the frame sets of the top three clusters to estimate the affinities in different binding modes.

**FIGURE 5:**
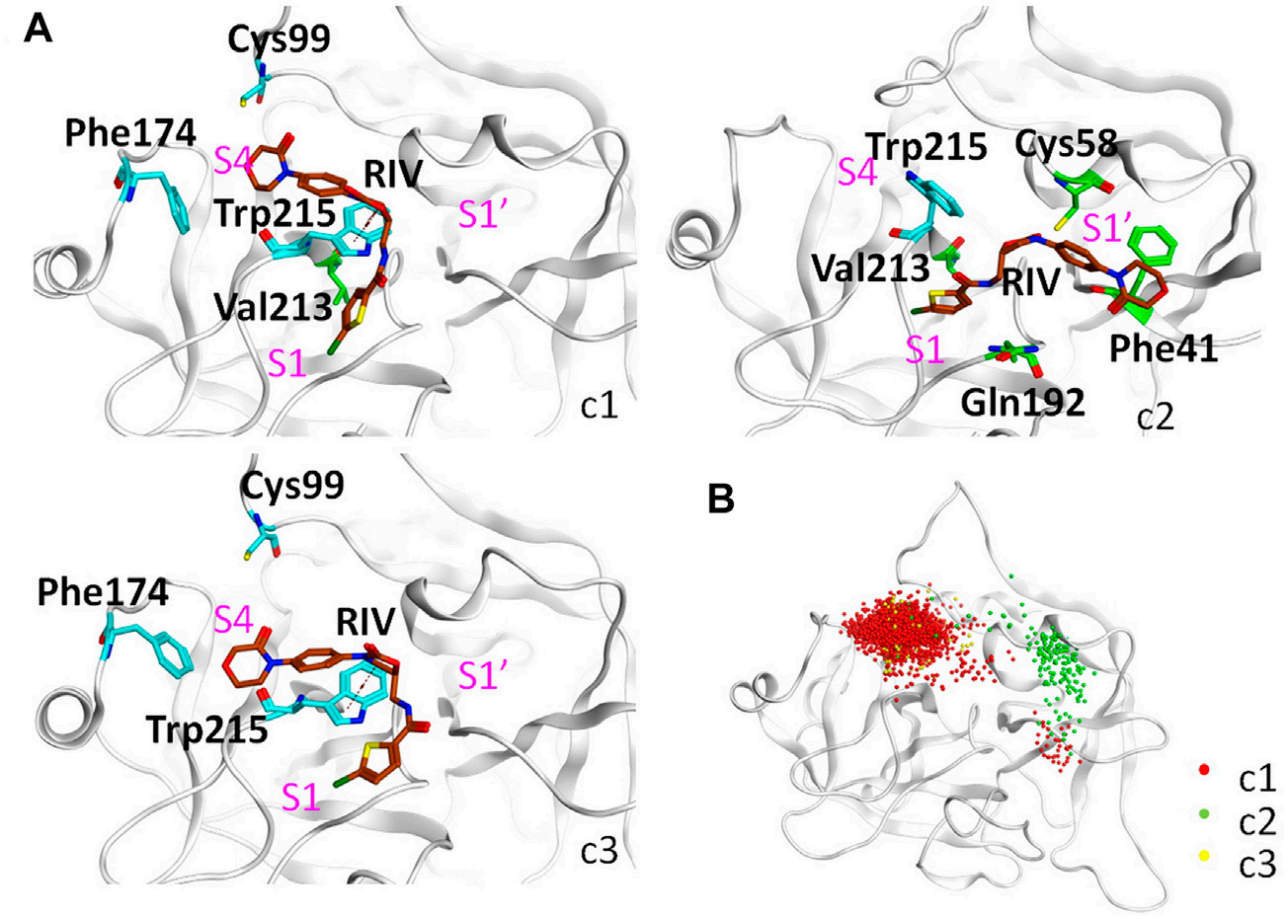
Detailed analysis on the binding of RIV in the Y99C mutant. **(A)** Representative binding modes of the top three clusters. RIV carbon atoms are colored in brown, the S4 pocket residues are shown in cyan, other residues important to RIV binding are shown in green, and the positions of the three pockets are labeled in magenta. **(B)** Distributions of the R4 group are visualized by the positions of the C3 atom of RIV.

As shown in Figure 5A and Table 4, the top three clusters have already exhibited alternative binding patterns. The top cluster (c1) occupies 83% of the population, where the R1 and R4 group of RIV still stay around the S1 and S4 pocket, respectively. However, this binding pattern is quite dynamic and not as stable as that in the rigid S1S4 binding in the crystal structure of wild type FXa. A major difference is the flip of the side chain of Trp215 between the S4 and S1 pocket, which not only reshapes the aromatic cage of the S4 pocket but also brings a shift of RIV outward by the hydrophobic interactions between the rings of Trp215 and the R2 & R3 rings of RIV. This shift gives RIV more flexibility to interact with surrounding residues with different binding poses, such as the hydrophobic interactions with Val213 and hydrogen bonds with His57 and Ser195, which may compensate some of the loss of binding energy in the S4 pocket. The second cluster (c2) is of only 7% of all the simulation time, in which RIV is relatively stable bound in a totally unexpected binding pattern, with R1 in the S1 pocket and R4 in the S1’ pocket. In this pattern, RIV totally loses its interaction with Phe174 and Cys99 but still maintains hydrophobic interactions with Trp215 and Val213 in the S1 pocket and gains additional interactions from the residues in the S1’ pocket, such as Phe41, Cy58, and Gln61. However, the S1’ pocket is relatively exposed to the solution, making it easy for RIV to move away. At last, the third cluster (c3) is of only 4% of the total sampling, whose representative structure is somewhat like the stable S1S4 binding in wild type, but Trp215 is flipped and R4 of RIV is more likely sandwiched between the rings of Phe174 and Trp215. According to the MM-PBSA calculations on the concatenated trajectory of Y99C mutant, the c1 (−96.505 ± 0.454 kJ/mol) and c3 (−83.085 ± 1.847 kJ/mol) show relatively stronger binding affinity than c2 (−72.622 ± 1.554 kJ/mol) featured as the S1S1’ binding pose (Supplementary Table S3).

**TABLE 4:** Top three binding modes in the Y99C mutant.

	R1	R4	Frequencies (%)	Total binding energy	Key residues	Residue contributions
				FX:RIV	(Contribution ≤ −2 kJ/mol)	(kJ/mol)
c1	S1	S4	82.77	−96.505 ± 0.454	CYS-99	−3.4787
					PHE-174	−4.8023
					VAL-213	−2.3387
					TRP-215	−10.0666
c2	S1	S1′	7.06	−72.622 ± 1.554	PHE-41	−2.2875
					CYS-58	−2.0859
					GLN-192	−2.0565
					VAL-213	−2.7228
					TRP-215	−6.3439
c3	S1	S4	4.33	−83.085 ± 1.847	CYS-99	−2.2025
					PHE-174	−3.4795
					TRP-215	−10.5807

Binding modes are based on the representative structures of the top three clusters of the Y99C trajectories, where R1 and R4 refer to the positions of the chlorothiophene and the morpholinone rings of RIV, and S1, S1’, and S4 refer to the RIV groups that are roughly close to the positions of the corresponding FXa pockets. Sampled conformations belonging to c1, c2, and c3 were picked out for MM-PBSA, calculations of the RIV, and binding energy, respectively. The key contributed residues and their contributions of each cluster are also listed in the last two columns of the table.

It should be noted that a single representative structure of the clusters may not fully describe the dynamics in different binding poses of RIV. Therefore, the distribution of the R4 group (Figure 5B) was compared by superposition of the coordinates of RIV’s C3 atom. In the first cluster, although R4 stays in the S4 pocket for the most time, it may move a little toward the catalytic pocket or even far away to the S1’ pocket. In the second cluster, the distribution of R4 mainly assembles in the S1’ pocket, with only minor frames diffused into the S4 pocket. At last, the distribution of the R4 group is more concentrated in S4 of the third cluster.

Possibly because of the variations between the trajectories of Y99C simulations, the difference in binding pattern is more obvious if we clustered the three repeats separately with a lower cut-off (Supplementary Figure S6). In the first trajectory Y99C_I, although there is some deformation of the S4 pocket, most of the time R4 stays around S4 to interact with Phe174 or the residues His57, Cys58, or Tyr60 close to the catalytic pocket. At the same time, the flip of Trp215 to the S1 pocket destabilizes the binding or R1 in the S1 pocket. In the second trajectory, most of the time the side chain of Trp215 is not flipped; thus, R1 can stay stably in the S1 pocket. However, the ring of R4 loses its hydrophobic interaction with Phe174 and has an astonishing shift toward the catalytic pocket or even to the S1’ pocket, although most of the time, the electrostatic interaction with the thiol of Cys99 is kept, and the distribution of R4 is quietly diffused around S4 in cluster one (c1) or around S1’ in c2 & c3. As a result, the binding pattern is relatively shifted from the typical S1S4 to the atypical S1S1’. At last, in the third trajectory, the S4 pocket is stronger and relatively stable, and the binding of RIV is quite similar to the typical S1S4 binding in wild type.

## Discussion

To explain the unexpected results of the Y99C mutant, the molecular dynamics simulations in this work provide a great help to explore the microscopic details of RIV-FXa binding in the dynamic condition of dilute solution, although we are aware that limited resources of computations and requirements of accuracy may lead to some deviations from reality.

At first, the great flexibilities in both the protein and the drug indicate that our analyses are not enough to sufficiently reflect the whole landscape of the dynamic binding. On one side, the flexibilities may be the reason for RIV in equilibration of multiple binding poses, such as the fluctuation of the 99-loop and the flipping of Trp215’s side chain. On the other side, they made it quite complex to compare, analyze, summarize, and visualize the binding in an easy and intuitive manner because the shape and the positions of the drug and the binding pockets are always changing. For example, RIV is an extended flexible compound with four rings, where the chlorothiophene (R1) and the morpholinone (R4) rings tend to bind with different pockets somewhat independent to each other. In structural clustering, the RMSD of all atoms of RIV may include all the structural information, but the distribution of different binding patterns with different pockets could not be clearly visualized by the superposed mass center of whole RIV. Considering that the R1 group should be more stably bound as discussed later, we chose to focus on the more dynamic distribution of the R4 group by superposition of the coordinates of the C3 atom on it. Conversely, a similar NOAC apixaban is much more rigid and compact, making it easier to be described, but at the same time, it may lose some possibility to bind FXa in alternative patterns as RIV, which may partially explain its lower binding affinity in some mutants, as discussed earlier (Qu et al., 2019). From this example, we would again emphasize the recognition of the important influence of the flexibility of both the target protein and the drug molecule, which was often neglected in structure-based drug design and screening using static crystal structures but more and more considered in recent studies, especially in protein and antibody design (Corbeil et al., 2021).

Second, here, we used a popular method of clustering to find a representative structure of different binding patterns for illustration. However, the representative structures may not always describe the difference between the bindings very well since the results of clustering may be importantly affected by the distribution of samples. Due to the limited resources, we chose to repeat the simulations thrice for 200 ns each instead of an extended simulation to obtain more diffused sampling. However, the sampling in the three trajectories of the Y99C mutant is quite distinctive with each other, as described previously. For comparison with wild type and Y99A, we used the clustering on all samples of the three trajectories combined together, which not only enlarged the pool of samples but also the variations within one cluster. Therefore, we supplemented the clustering of separate trajectories so as to visualize the different binding patterns more clearly. Adoption of alternative algorithms or strategies of clustering may improve the recognition of different binding patterns (Fraley and Raftery, 2002; Xu and Wunsch, 2005) but may also introduce more artificial bias or differences with earlier analyses.

At last, another source of error may come from the force field that we used. The simulations on the three FXa systems were performed as early as 2018, using the popular CHARMM36 force field for FXa, TIP3P model for water, and CGenFF to generate parameters for RIV as we did not evaluate the effect of force field using alternative ones. However, according to our discussions with experts in MD simulations, we would expect some improvement in the validity of our simulations if some conditions could be applied further. First, the general CHARMM36 force fields in GROMACS were fit for folded structures. However, according to the results of our simulations and earlier studies (Wang et al., 2012; Qu et al., 2019; Qu and Xu, 2019), the region of the 99-loop and the 174-loop may have much higher flexibilities in dilute solution rather than those of a compact aromatic cage as in the crystallization condition, especially when the hydrophobic environment of the S4 pocket is somewhat damaged by mutations. Therefore, if the loop regions were described by force fields specifically adjusted for an intrinsic disordered region, such as a folded-IDP balanced force field ff03CMAP that we used recently (Zhang et al., 2019; Mu et al., 2022), the dynamics of the critical loop regions might be more realistic, especially when we did not have enough resources for fully sufficient equilibrations. Second, the point charge generated by CGenFF for the chloride of RIV is too unsophisticated to describe the anisotropy of the electron density around it, which may form a σ-hole leading to a cation-π interaction with Tyr228 or a halogen bond with the conserved Asp189 deep in the bottom of the S1 pocket. The halogen bond might provide one of the reasons for the chlorothiophene as a critical pharmacophore for the specific binding of RIV in FXa. Although, in recent years, the importance of halogen bond has been highly recognized in biology and drug design, and great advances has been made to apply it in simulations, the cases in classical MD simulations are still too limited for us to find a way to integrate it into our simulations successfully (Franchini et al., 2018; Zhu et al., 2019; Dong et al., 2020; Zhang et al., 2020; Zhu et al., 2020). The inaccurate description of the chloride interactions may underestimate the binding of RIV in the S1 pocket, leading to overestimation of clusters with R1 out of the S1 pocket. However, this deviation happens in both wild type and the mutants and may not directly affect the deformation of the S4 pocket and the shift of R4 to the S1’ pocket. Therefore, in our opinion, the qualitative conclusion on the dynamic binding of RIV in the Y99C mutant should be still valid.

## Conclusion

In this work, we report a novel mutant of coagulation factor X, Tyr319Cys (Y99C of FXa), identified in a patient with severe bleeding, which can bind and cleave specific chromogenetic substrates at a normal level. Consistently, molecular dynamics simulations confirmed that its S4 pocket was much less deformed than that of the Y99A mutant and may maintain the binding affinity with rivaroxaban through dynamic binding between multiple poses. Detailed structural analyses indicated that the backbone of the 99-loop is only in minor fluctuation compared with Y99A and kept part of the hydrophobic and H-bonds with the S4 pocket. At the same time, the flexible flipping of Trp215’s side chain may help stabilize alternative binding poses with the R4 group in the catalytic pocket or even in the S1’ pocket. This result again emphasizes the importance to consider the flexibilities of both target protein and drug compound in structure-based drug design and may support the hypothesis to develop similar recombinant FXa as a potential reversal agent for novel oral anticoagulants.

## Data Availability

The original contributions presented in the study are included in the article/Supplementary Material, further inquiries can be directed to the corresponding authors.
