# Supplementary figures and images for: Unexpected Dynamic Binding May Rescue the Binding Affinity of Rivaroxaban in a Mutant of Coagulation Factor X
Source: Front Mol Biosci. 2022 May 5;9:877170. doi: 10.3389/fmolb.2022.877170 (PMC9117642; doi:10.3389/fmolb.2022.877170)

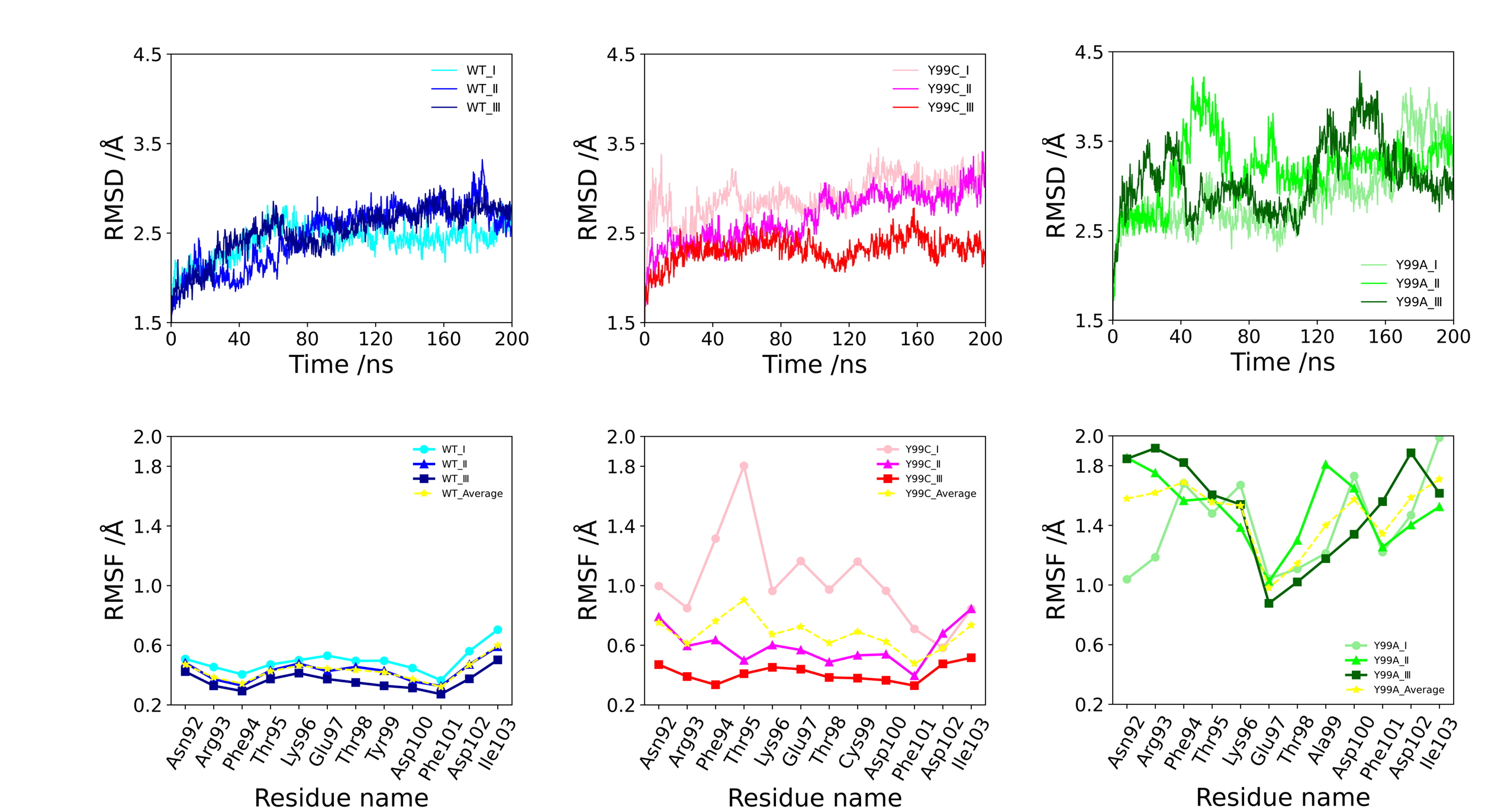

Supplement: Supplementary file 1 [file Image3.JPEG]

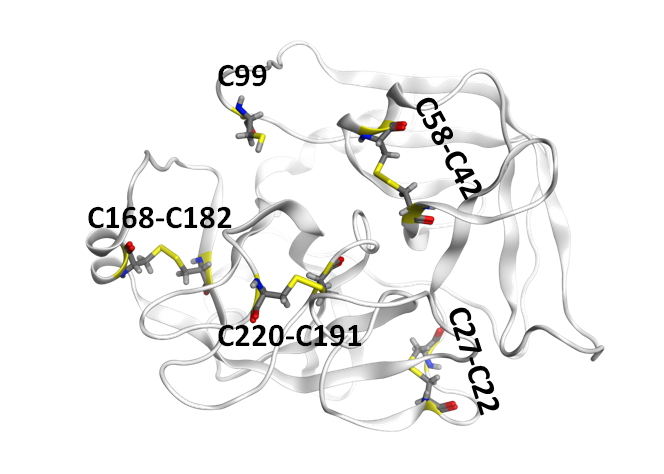

Supplement: Supplementary file 2 [file Image1.JPEG]

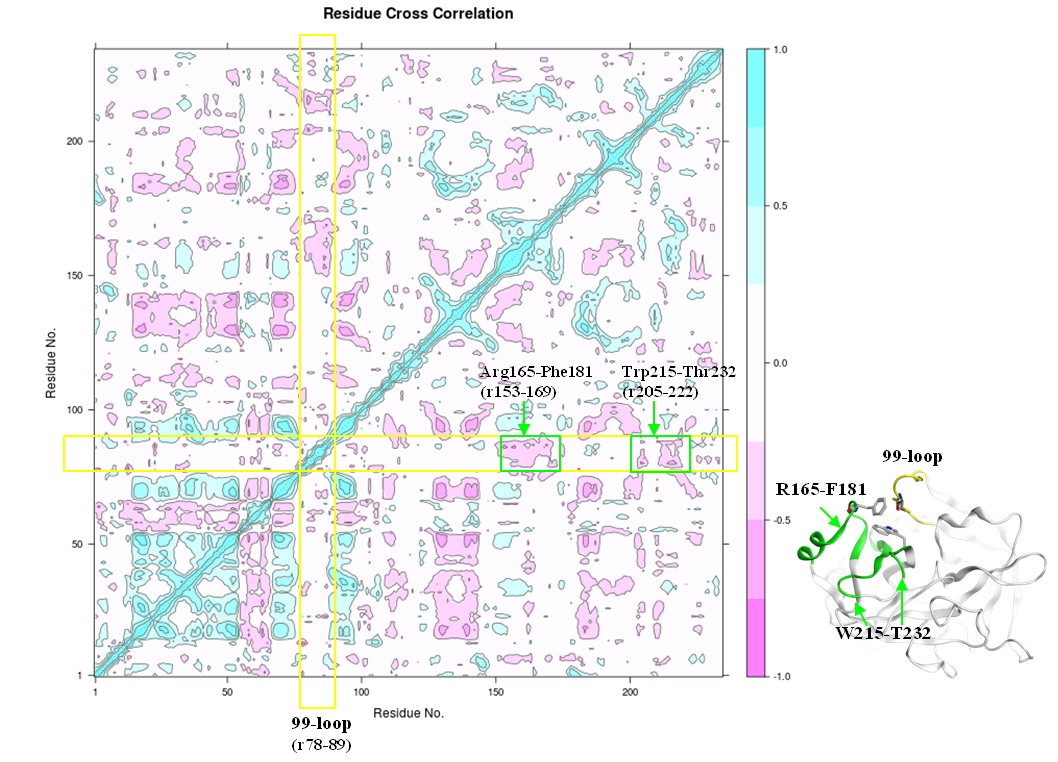

Supplement: Supplementary file 3 [file Image4.JPEG]

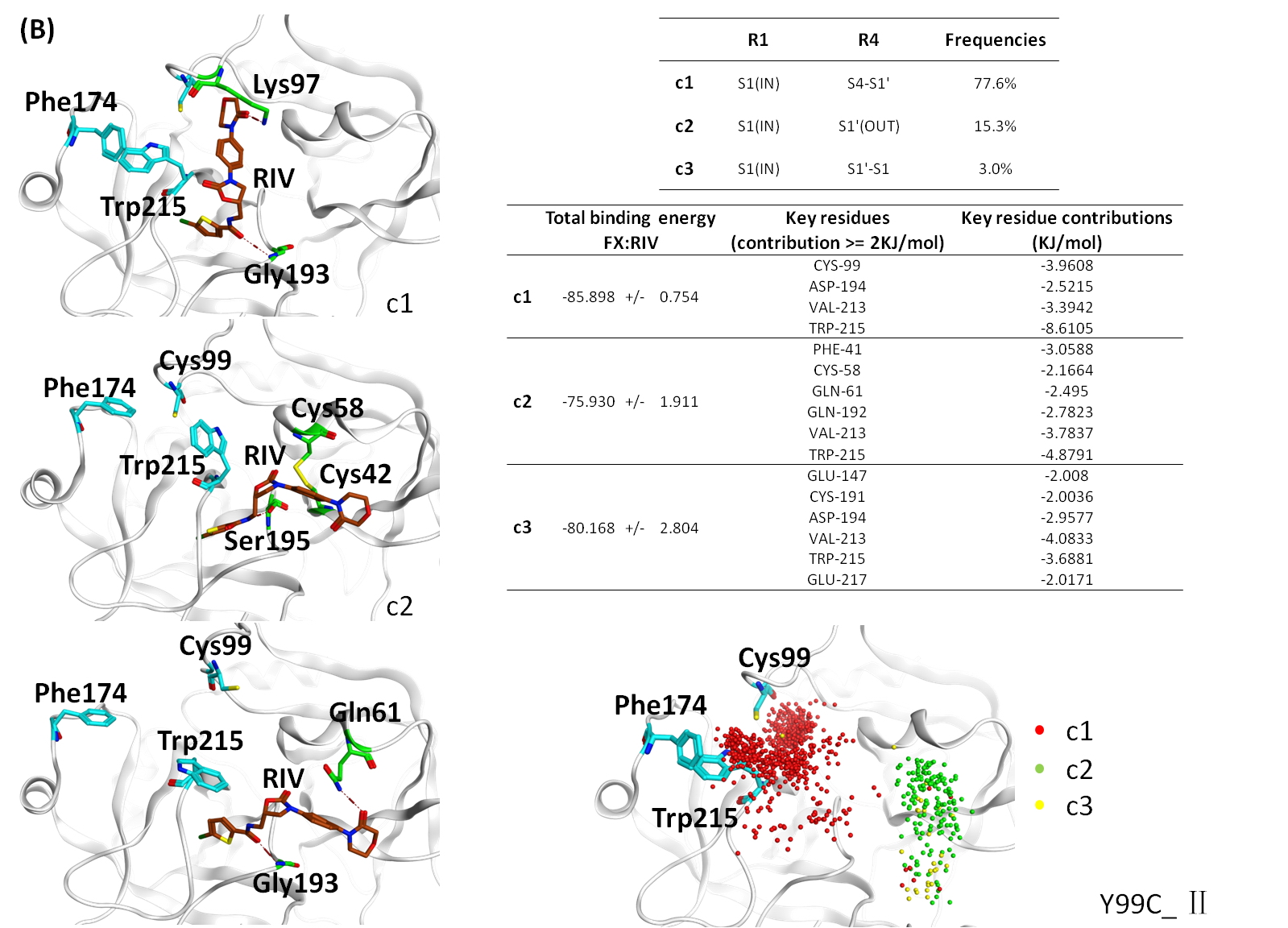

Supplement: Supplementary file 5 [file Image7.JPEG]

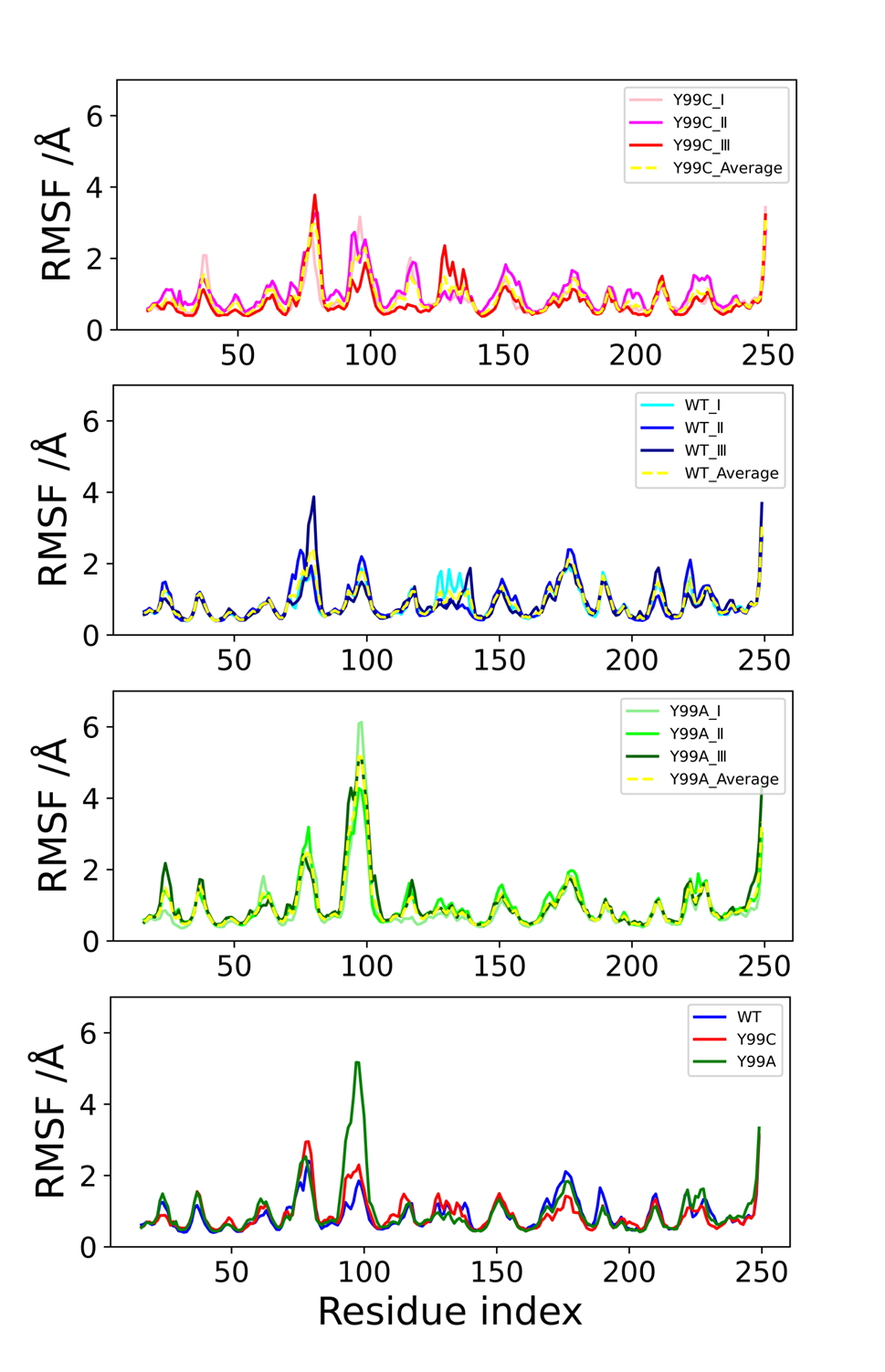

Supplement: Supplementary file 6 [file Image2.JPEG]

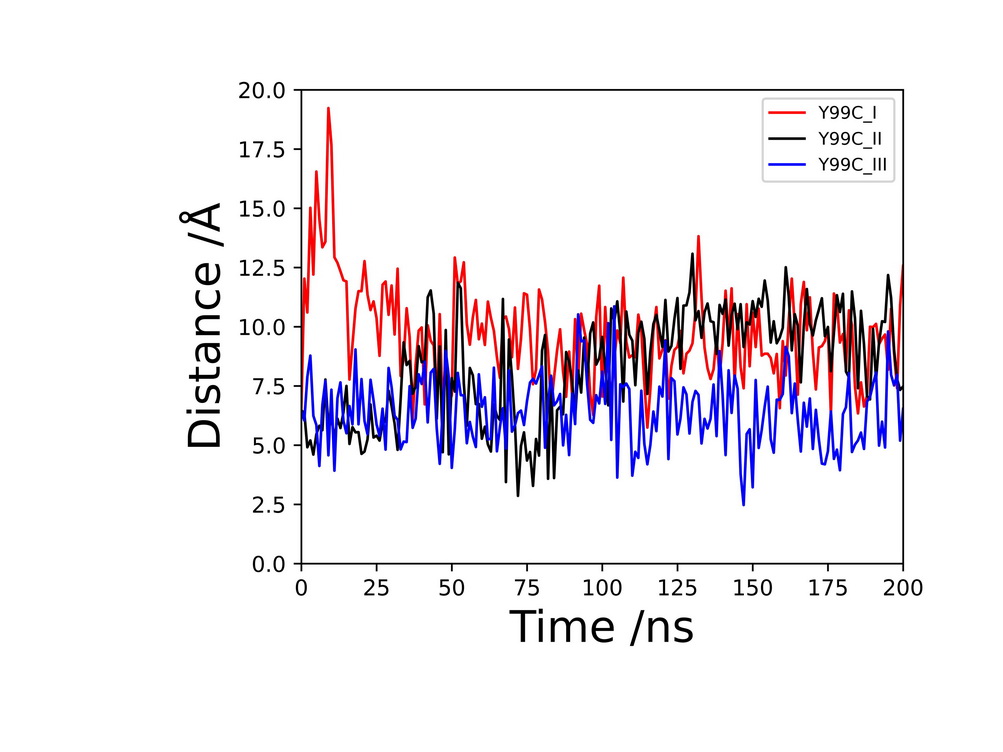

Supplement: Supplementary file 7 [file Image5.JPEG]

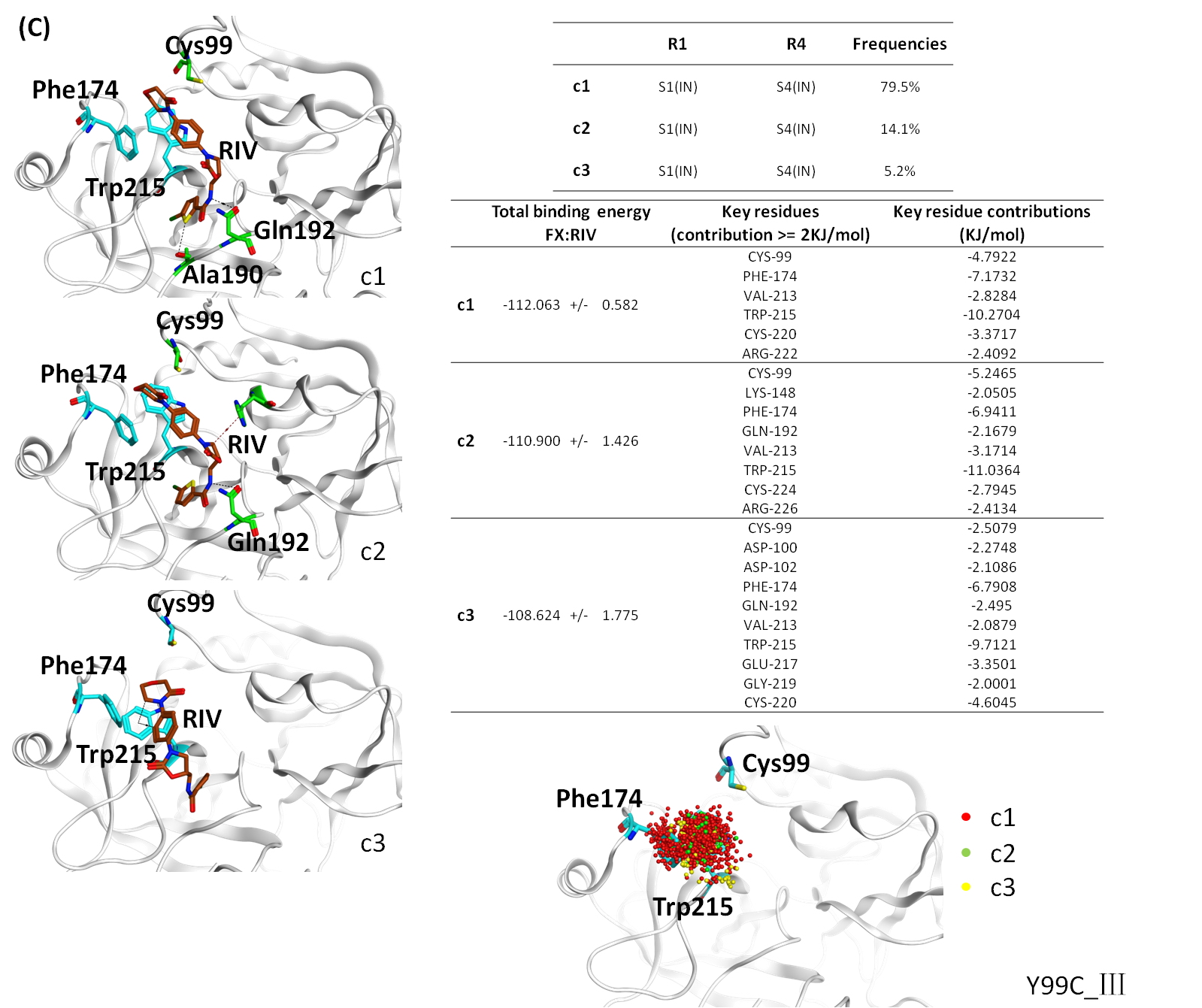

Supplement: Supplementary file 9 [file Image8.JPEG]

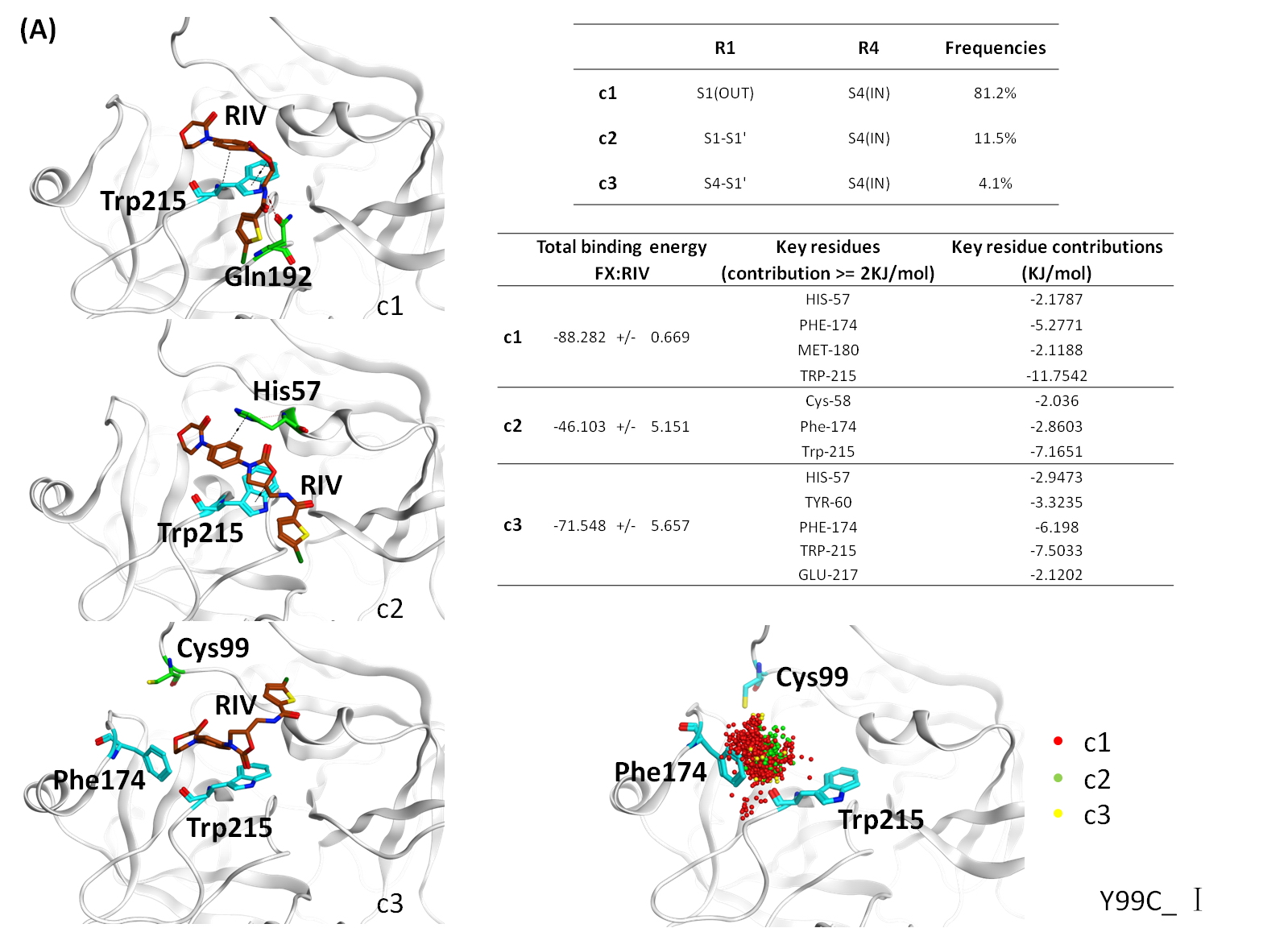

Supplement: Supplementary file 10 [file Image6.JPEG]
