# Supplementary material for: Unexpected Dynamic Binding May Rescue the Binding Affinity of Rivaroxaban in a Mutant of Coagulation Factor X
Source: Front Mol Biosci. 2022 May 5;9:877170. doi: 10.3389/fmolb.2022.877170 (PMC9117642; doi:10.3389/fmolb.2022.877170)
Supplement: Supplementary file 8 [file DataSheet2.docx]

Unexpected dynamic binding may rescue the binding affinity of Rivaroxaban in a mutant of coagulation factor X

Zhi-Li Zhang^1†^, Chen-Chang Ming^2†^, Si-Ying Qu^1^, Qiulan Ding^2,3*^ and Qin Xu^1*^

^1^State Key Laboratory of Microbial Metabolism & Joint International Research Laboratory of Metabolic and Developmental Sciences, School of Life Sciences and Biotechnology, Shanghai Jiao Tong University, Shanghai, P.R. China

^2^Department of Laboratory Medicine, Ruijin Hospital, Shanghai Jiao Tong University School of Medicine, Shanghai, China

^3^Collaborative Innovation Center of Hematology, Shanghai Jiao Tong University School of Medicine, Shanghai, China

† **These authors contributed equally to this work.**

*** Correspondence:**xuqin523@sjtu.edu.cn (Q.X.); qiulan_ding@shsmu.edu.cn (Q.D.)

# Supplementary Figures and Tables

**Table S1.** The contributions of the key residues in the S4 pocket to the binding of RIV. The detailed contributions of the key residues 99 (Tyr99 in wild type, Cys99 and Ala99 in the mutants), Phe174 and Trp215 to the binding of RIV are compared between the three systems.

|  | **Residue** | **MM** | **Polar Solvation** | **SASA** | **Total** |
| --- | --- | --- | --- | --- | --- |
| **WT** | **TYR-99** | -5.919±0.261 | 3.291±0.178 | -0.571±0.023 | -3.205±0.149 |
|  | **PHE-174** | -4.831±0.209 | 1.336±0.068 | -0.489±0.024 | -3.985±0.176 |
|  | **TRP-215** | -13.214±0.340 | 7.705±0.236 | -0.937±0.028 | -6.453±0.178 |
| **Y99C** | **CYS-99** | -3.778±0.126 | 1.240±0.056 | -0.382±0.013 | -2.916±0.100 |
|  | **PHE-174** | -4.702±0.176 | 1.324±0.055 | -0.574±0.021 | -3.947±0.147 |
|  | **TRP-215** | -15.833±0.313 | 8.446±0.178 | -1.101±0.023 | -8.497±0.174 |
| **Y99A** | **ALA-99** | -0.070±0.021 | 0.056±0.022 | -0.008±0.004 | -0.022±0.017 |
|  | **PHE-174** | -0.216±0.056 | 0.087±0.023 | -0.027±0.007 | -0.156±0.043 |
|  | **TRP-215** | -2.224±0.229 | 1.150±0.120 | -0.174±0.017 | -1.244±0.128 |

**Table S2.** The possible H-bonding residues with RIV in the three systems, with occupancy≥2%, distance≤3.5Å, angle≤30º.

| **Systems** | **Residues** | **Occupancies** |
| --- | --- | --- |
| **WT** | **Gly216** | 15.0% |
|  | **Gly219** | 13.6% |
|  | **Gln192** | 10.6% |
|  | **Phe174** | 8.6% |
|  | **Trp215** | 6.6% |
|  | **Ser173** | 4.8% |
|  | **Arg143** | 4.8% |
|  | **Glu217** | 4.4% |
|  | **Gln61** | 3.6% |
|  | **Tyr99** | 2.8% |
|  | **His57** | 2.4% |
|  | **Ser195** | 2.1% |
| **Y99C** | **Gly193** | 21.6% |
|  | **Gln192** | 19.9% |
|  | **Gly216** | 14.4% |
|  | **Ser195** | 8.2% |
|  | **Lys96** | 5.8% |
|  | **His57** | 5.3% |
|  | **Ser214** | 3.9% |
|  | **Gln61** | 2.6% |
|  | **Trp215** | 2.5% |
|  | **GLY219** | 2.4% |
|  | **Glu217** | 2.0% |
| **Y99A** | **Lys62** | 8.1% |
|  | **Gly219** | 5.4% |
|  | **Gln192** | 5.3% |
|  | **Gln61** | 2.7% |
|  | **Gly216** | 2.4% |


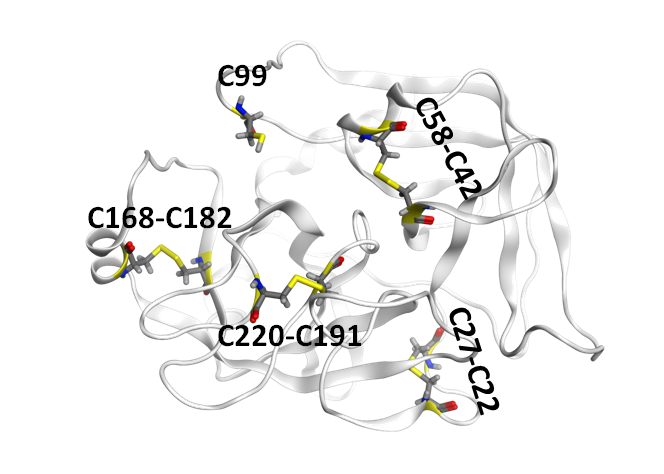


**Figure S1.** The model for Y99C mutant of coagulation factor X generated by H++. Protonation states for Cysteine residues in the mutant were calculated by the H++ web server. Salinity, internal dielectric and external dielectric conditions were set in default. The protein was protonated at an assuming PH 7.0.


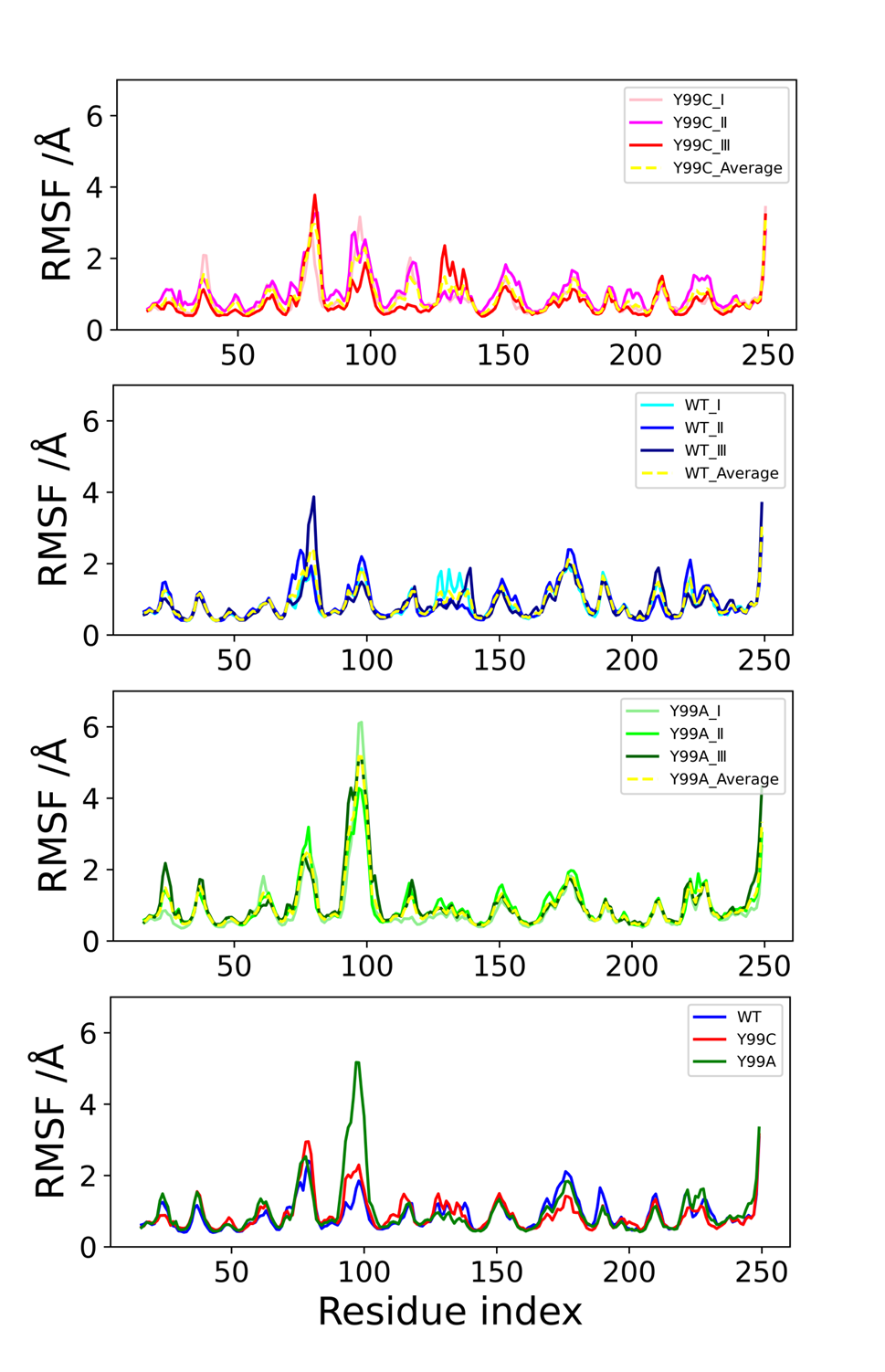


**Figure S2.** Fluctuations of the whole protein backbone in the wild type (WT), Y99C and Y99A mutants of coagulation factor X with RIV bounded, with the three trajectories compared with their averages in the upper three panels and the averages of the three systems compared in the bottom panel.


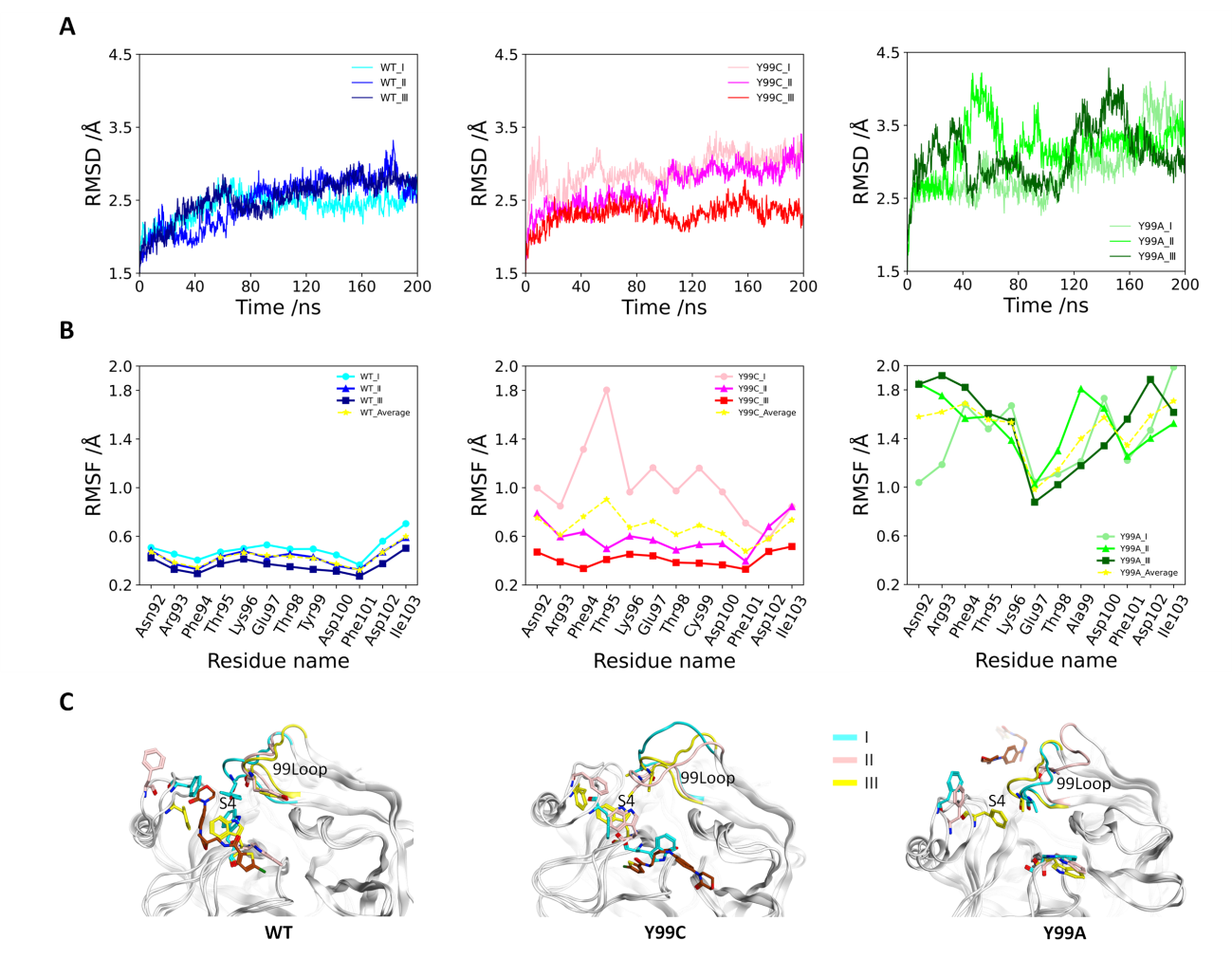


**Figure S3.** Fluctuations of the backbone of the 99-loop in the wild type (WT), Y99C and Y99A mutants of coagulation factor X with RIV bounded. (A) Comparison of the backbone RMSD between the three trajectories of each system. The backbone of the 99-loop in Y99C is not as consistent as in WT, but still relatively stable, which may suggest multiple folding conformations. On the contrary, the 99-loop in Y99A is neither converged nor stable, suggesting it quite unfolded into random coil. (B) The RMSF of the residues on the 99-loop in the three trajectories, WT(blue), Y99C(red) and Y99A(green), with their average shown in yellow.


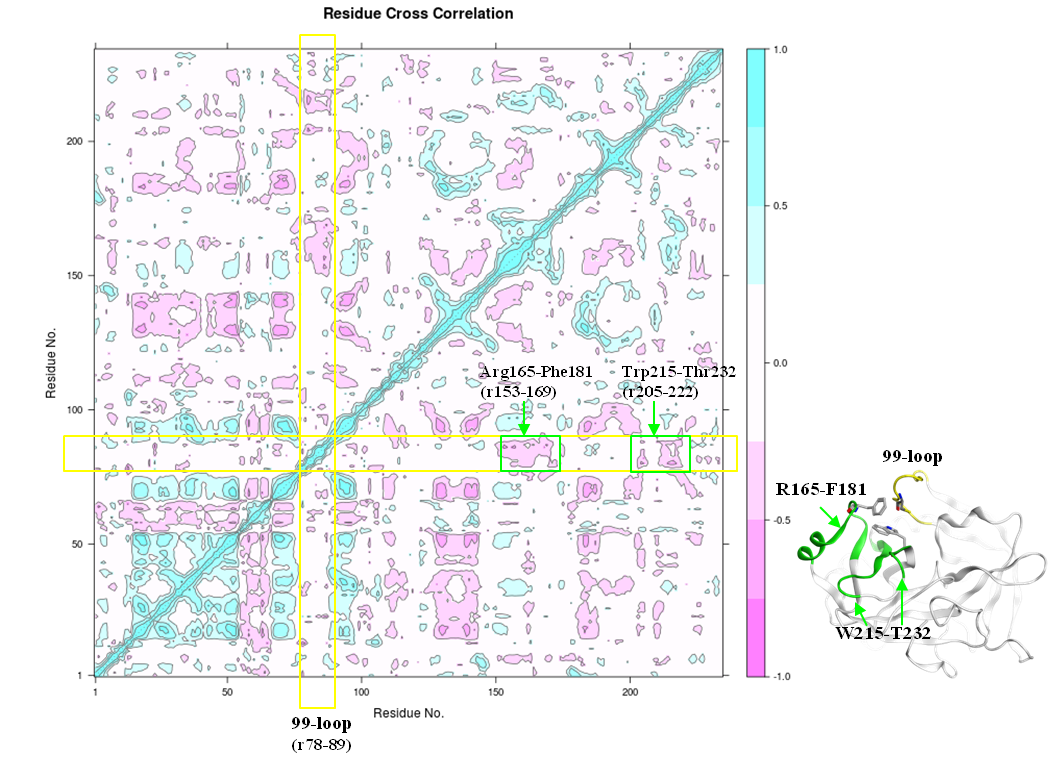


**Figure S4. Residue cross correlation analysis for Cα atoms of the FX Y99C mutant.** The residues in chymotrypsin numbers from the structure 2W26 were sequentially renumbered from 1 to 234. The region of the 99-loop (78-89) was highlighted in the box. Two segments showing significant correlations with 99-loop were in green box, with their ribbons colored in green in the structure correspondingly.


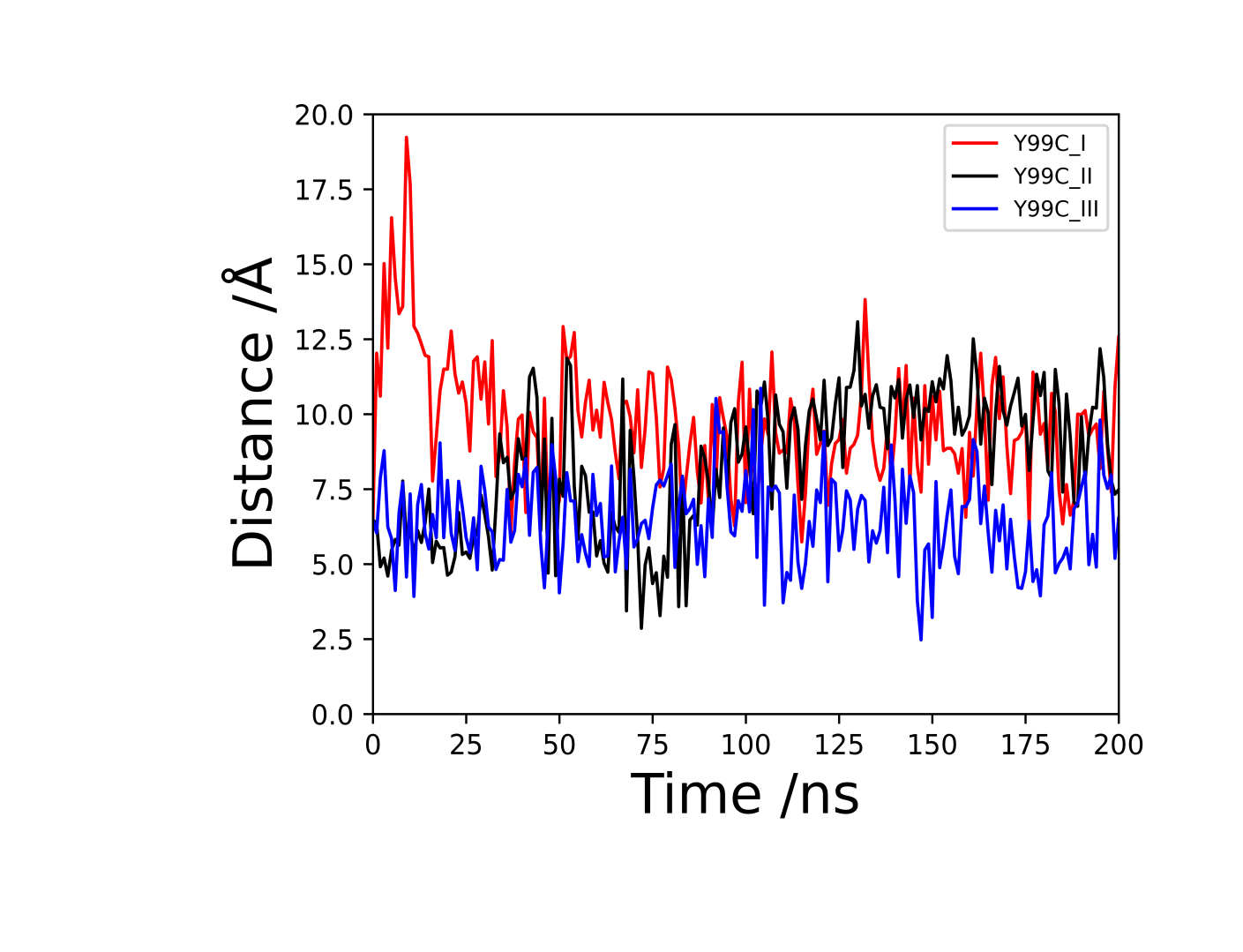


**Figure S5**. Time evolution of the distance between H57[ND1] and C99[HG1] in the three repeats of 200ns MD simulations of Y99C mutant. The average distance is 9.7, 8.4 and 6.4 Å in Y99C_I, Y99C_II and Y99C_III respectively. All of them are too far for a typical hydrogen bond.


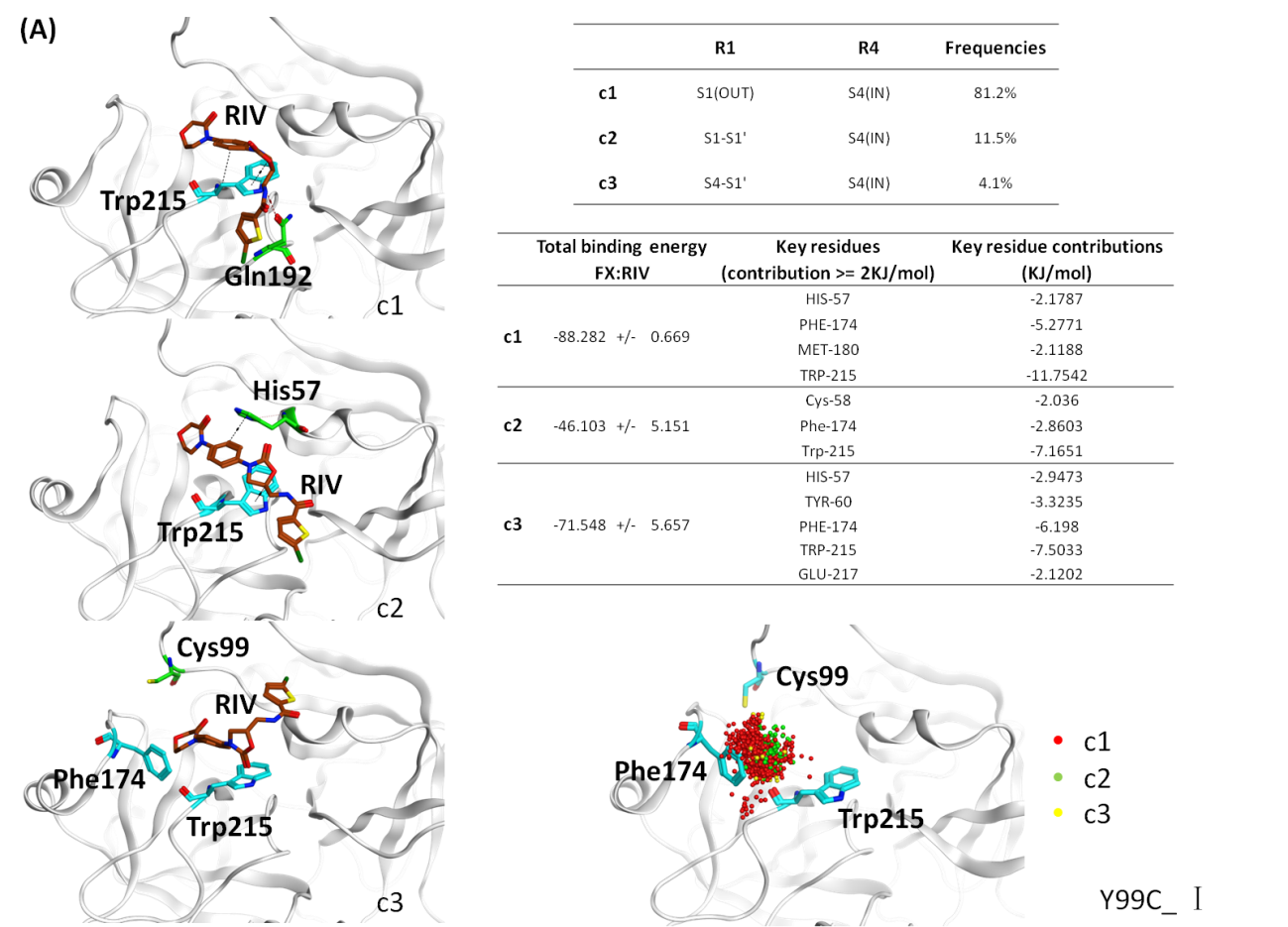


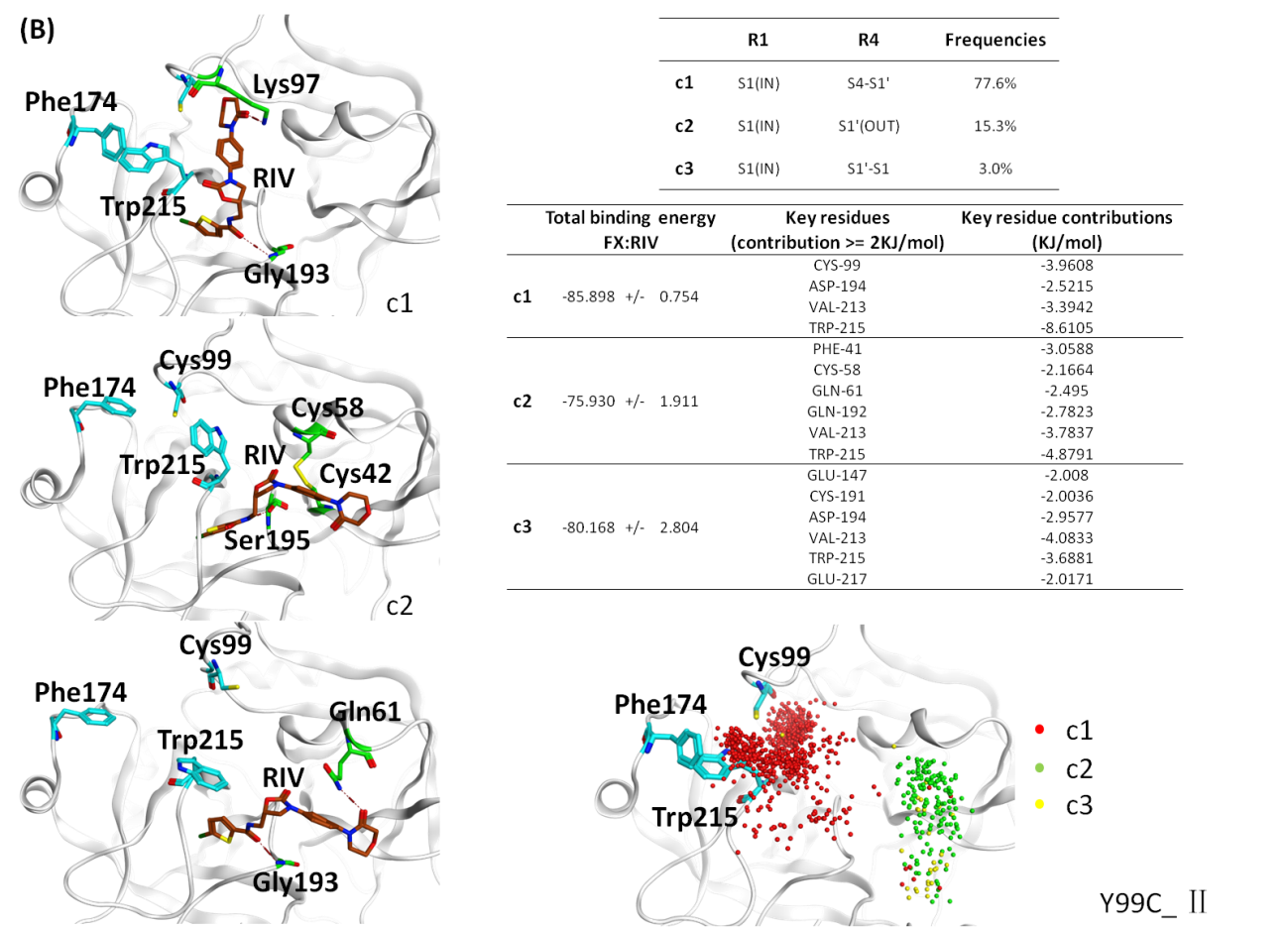


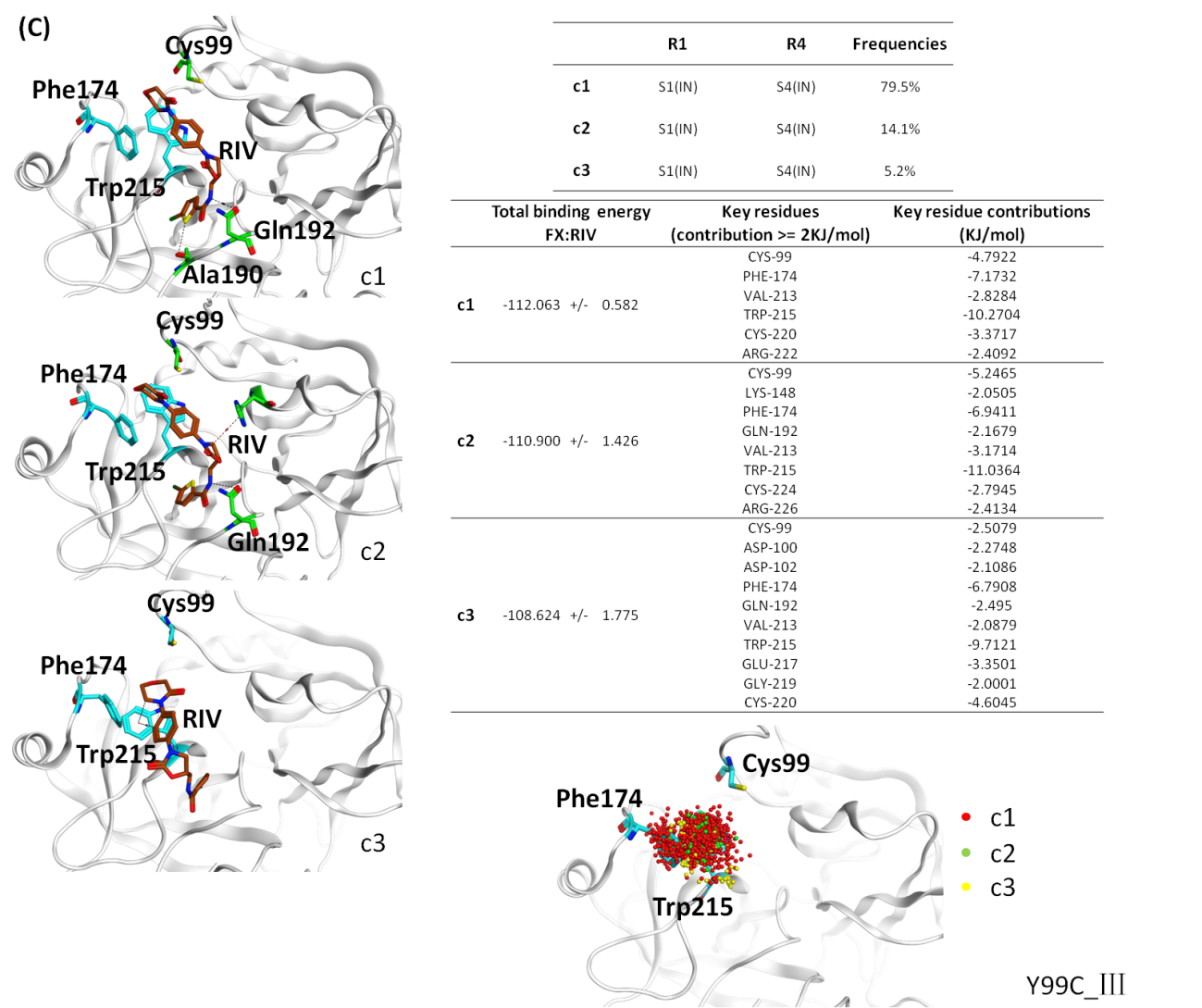


**Figure S6**. Detailed analysis on the binding of RIV in Y99C mutant. The representative binding modes of the first three clusters are illustrated in 3D structure (on left), with the positions of the R1 and R4 groups of RIV (upper on right), the energetic contributions of key residues (middle on right), and the distributions of R4 group analyzed. (A) The Y99C_I trajectory. (B) The Y99C_II trajectory. (C) The Y99C_III trajectory.
